# SDCCAG3 inhibits adipocyte hypertrophy and improves obesity-related metabolic disorders via SDCCAG3/SMURF1/PPARγ axis

**DOI:** 10.1016/j.jlr.2025.100772

**Published:** 2025-03-07

**Authors:** Fenglei Huo, Chenghang Liu, Xi Wang, Jinzheng Li, Zhifeng Wang, Duanqin Liu, Weipeng Lan, Xingyan Zhu, Jing Lan

**Affiliations:** 1 Department of Prosthodontics, School and Hospital of Stomatology, Cheeloo College of Medicine, Shandong University & Shandong Key Laboratory of Oral Tissue Regeneration & Shandong Engineering Research Center of Dental Materials and Oral Tissue Regeneration & Shandong Provincial Clinical Research Center for Oral Diseases, Jinan, China; 2 College of Traditional Chinese Medicine, University of Traditional Chinese Medicine, Jinan, China; 3 Department of Pediatric Dentistry, School and Hospital of Stomatology, Cheeloo College of Medicine, Shandong University & Shandong Key Laboratory of Oral Tissue Regeneration & Shandong Engineering Research Center of Dental Materials and Oral Tissue Regeneration & Shandong Provincial Clinical Research Center for Oral Diseases, Jinan, China; 4 Department of Oral and Maxillofacial Surgery, School and Hospital of Stomatology, Cheeloo College of Medicine, Shandong University & Shandong Key Laboratory of Oral Tissue Regeneration & Shandong Engineering Research Center of Dental Materials and Oral Tissue Regeneration & Shandong Provincial Clinical Research Center for Oral Diseases, Jinan, China

**Keywords:** adipose tissue, cell signaling, glucose, insulin resistance, metabolic disorders, obesity, PPARγ, SDCCAG3, SMURF1

## Abstract

Obesity is a prevalent global disease associated with various metabolic disorders. The expansion of white adipose tissue plays a pivotal role in regulating obesity-related metabolic dysfunctions. This study identified serum-defined colon cancer antigen 3 (SDCCAG3) as a novel key modulator of adipocyte metabolism. In adipose-specific SDCCAG3 knockout mice fed a high-fat diet, pathological expansion of adipose tissue, impaired glucose tolerance, insulin resistance, increased inflammatory markers, and augmented hepatic lipid accumulation were observed. Conversely, obesity models by specific overexpression of SDCCAG3 in adipose tissue confirmed that SDCCAG3 alleviated pathological expansion of adipose tissue, improved obesity-related metabolic disorders, with no observed changes in adipose tissue development under normal dietary conditions. Mechanistically, SDCCAG3 enhanced the stability of peroxisome proliferator-activated receptor gamma (PPARγ) by preventing its degradation via the ubiquitin-proteasome system through the SMAD specific E3 ubiquitin protein ligase 1 (SMURF1). Additionally, SDCCAG3 was subjected to negative transcriptional regulation by PPARγ, forming a SDCCAG3-PPARγ-SDCCAG3 loop that enhanced adipocyte lipid metabolism. Collectively, these findings demonstrated that SDCCAG3 functioned as a beneficial positive regulator of adipose tissue expansion and metabolic homeostasis, indicating its potential as a therapeutic target for metabolic diseases associated with nutrient excess.

Obesity has emerged as both a disease and a global epidemic, affecting over one billion people (1). Its measurement helps estimating prevalence and mortality rates (2). Research indicates that obesity significantly triggers various diseases, including diabetes, atherosclerosis, hypertension, and several cancers, as well as being associated with chronic inflammation (3, 4, 5, 6, 7).

In obesity, white adipose tissue (WAT), as the main organ regulating energy balance, expands through adipocyte hypertrophy or hyperplasia (8, 9). Enlarged adipocytes are linked to systemic insulin resistance and inflammation from increased pressure and hypoxia (10, 11). Exceeding the storage capacity of adipocytes can lead to dysfunction and metabolic disorders, influencing the classification of individuals as either metabolically healthy or unhealthy obese (12). Adipocyte size is crucial in addressing obesity-related metabolic disorders, emphasizing the importance of limiting adipose tissue expansion for metabolic health (13, 14).

PPARγ dysfunction is characteristic of hypertrophic obesity. This condition relies on PPARγ as a key transcription factor that regulates glucose homeostasis and insulin responsiveness, thereby affecting lipid metabolism and maintaining energy balance (15, 16, 17). During a high-fat diet, the activation of adipose tissue PPARγ can reduce adipocyte hypertrophy and improve metabolic function, while inhibiting PPARγ can exacerbate adverse metabolic effects (18, 19). The ubiquitin-proteasome system plays a crucial role in regulating protein stability (20). Ligand binding induces the degradation of PPARγ via the ubiquitin-proteasome system, inhibiting its transcriptional activity. In adipocytes, several ubiquitin-protein E3 ligases capable of degrading or stabilizing PPARγ have been identified, including F-box protein 9, tripartite motif-containing 25, and neural precursor cell expressed developmentally down-regulated proteins 4 and 8 (21, 22, 23, 24). In this study, we discovered that SDCCAG3 collaborated with the E3 ubiquitin ligase SMURF1 to attenuate the degradation of PPARγ, thereby promoting adipocyte metabolism.

SDCCAG3 is a protein associated with cilia formation and cell signaling, characterized by a coiled-coil domain. It plays a role in regulating cell motility and signal transduction, with functions closely linked to various biological processes, including cell mitosis, ciliogenesis, and protein transport (25, 26, 27, 28). For instance, mutations in SDCCAG3 are associated with genetic disorders such as polycystic kidney disease, which is often accompanied by ciliary dysfunction (27). Furthermore, SDCCAG3 is significant in regulating intracellular vesicle trafficking and cell division (26). Our previous research has also found that SDCCAG3 is differentially expressed in the bone tissue of hyperlipidemic rats and promotes osseointegration of implants (28). Those underscore SDCCAG3 as a significant target for investigating cellular functions and disease mechanisms. Regarding metabolism, a dual-system genetic methodology has identified SDCCAG3 as a potential shared gene candidate linked to both type 1 and type 2 diabetes (29). However, the functional implications of SDCCAG3 in adipose tissue and lipid metabolism remain unexplored.

In this study, our investigations revealed that SDCCAG3 functioned as a positive regulator of adipocyte activity. Through validation using both in vitro and in vivo experimental methodologies, we established that SDCCAG3 attenuated the degradation of SMURF1's substrate, PPARγ, thereby ameliorating lipid metabolic disorders. This finding demonstrated that SDCCAG3 serves as a beneficial factor for adipose tissue expansion and the regulation of metabolic homeostasis, positioning it as a potential therapeutic target for metabolic diseases in the context of nutrient surplus.

## Materials and methods

All animal experiments were conducted in accordance with the protocol approved by the Ethics Committee of Stomatology Hospital of Shandong University (NO.20221147). The use of experimental animals in this project complies with ethical guidelines.

### Animals

C57BL/6J mice were purchased from Beijing Vital River Laboratory Animal Technology Co., Ltd. and housed in Shandong Key Laboratory of Oral Tissue Regeneration. The mice were kept in individually ventilated cages (IVC) at a temperature of 22°C, with humidity ranging from 40% to 60%, a standard day/night lighting cycle, and ad libitum access to water and food. *Sdccag3*^*FLOX/+*^ and FABP4-Cre mice were purchased from Cyagen (Suzhou) Biosciences Co., Ltd. and bred to generate adipose-specific knockout mice (AKO-*Sdccag3*) with *Sdccag3*^*FLOX/FLOX*^ genotype and expression of the FABP4-Cre enzyme. Mice with *Sdccag3*^*FLOX/FLOX*^ genotype but lacking FABP4-Cre enzyme expression were used as controls (AKO-WT). The genotype of the mice was verified by PCR analysis of tail tissue, as shown in supplemental Table S1, which includes the guide RNA sequence. Six-week-old mice were utilized, and a high-fat diet comprising 60 kcal% fat (D12492, Research Diet) was administered to establish an obese mouse model. The control group was fed with a specific pathogen-free (SPF) mouse breeding and growth diet (Keao Xieli, Tianjin, China). There were five mice in each group.

### rAAV9-mediated specific overexpression of SDCCAG3 in adipose tissue

The rAAV9-FABP4p-MCS-*Sdccag3*-SV40 overexpression vector was purchased from Shanghai Genechem Co., Ltd. The virus was stored at −80°C and thawed on ice before use. It was diluted with sterile saline to achieve the virus titer 2E+11vg per mouse, and the injection site was disinfected with iodophor. Adipose tissue was infected by intraperitoneal injection, and the control group was injected with empty vector at the same virus titer. The injections were administered when the mice were 4 weeks old. Following the injections, feeding observations were conducted for two weeks, after which the mice were divided into a high-fat diet (HFD) and normal chow diet (NCD) groups for further feeding. There were six mice in each group.

### Micro-CT scanning imaging and reconstruction analysis

Micro-computed tomography (Micro-CT) was conducted using the Quantum GX2 Live Animal Imaging System (PerkinElmer) to scan the abdomen and lower limbs of mice at a resolution of 72 μm, generating DICOM files. These DICOM files were analyzed using Mimics Research 21.0. The distribution of visceral and subcutaneous fat in the abdominal and lower limb regions was assessed. The CT value of adipose tissue ranged from −187 to 22HU. The boundary of the abdominal cavity was used to differentiate between visceral fat (located within the abdominal cavity) and subcutaneous fat (located outside the abdominal cavity). The distribution of visceral and subcutaneous fat was evaluated in the sagittal plane (anatomical reference: penile bone), coronal plane (anatomical reference: bladder and testis), and horizontal plane (anatomical reference: external oblique muscle) using a split mask technique. Data analysis was performed by individuals who did not know the grouping of each mouse.

### Glucose tolerance test and insulin tolerance test

For the glucose tolerance test (GTT), mice were fasted for 16 h before the experiment while maintaining normal water intake. At the start of the experiment, the body weight of each mouse was recorded, and blood samples were collected from the tail vein using blood glucose test paper (Roche, Basel, Switzerland) and measured with a blood glucose meter (Roche). The baseline blood glucose level was recorded at 0 min. Following a 30-min acclimatization period, a 20% glucose solution was prepared with normal saline at a dosage of 2 g/kg, with an injection volume of 0.1 ml/g. The glucose solution was administered intraperitoneally. Blood glucose levels were subsequently measured and recorded at 15, 30, 60 and 120 min post-injection. For the insulin tolerance test (ITT), mice were fasted for 4 h before the experiment, while maintaining normal drinking water. Body weight was measured, and blood glucose levels were assessed before injection. The intraperitoneal injections of insulin were performed at a dosage of 0.5U/kg. Blood glucose levels were measured and recorded at 15, 30, 60, and 120 min after the injection. At the end of the experiment, each cage was supplemented with feed.

### Serum collection and biochemical analysis of mice

Mice were anesthetized by inhalation with isoflurane. Before blood coagulation, blood samples were collected from the inner canthal venous plexus using a capillary glass tube with an inner diameter of 0.3 mm. The samples were placed at 4°C overnight and centrifuged at 4000 rpm for 15 min to separate the serum. The serum was transferred to a new collection tube, packed and stored at −80°C for subsequent analyses. Serum triglyceride (TG), cholesterol (CHO), low-density lipoprotein cholesterol (LDLC), high-density lipoprotein cholesterol (HDLC), alanine aminotransferase (ALT) and aspartate aminotransferase (AST) were measured using commercial kits (S03030; S03040; S03027; S03042; S03025; S03029, Rayto) and an automatic biochemical analyzer (Chemray 800, Rayto). Serum free fatty acids (FFA) were quantified using a free fatty acid content detection kit (BC0590, Solarbio, Beijing, China). Serum insulin levels were determined using a mouse insulin ELISA kit (SEKM-0141, Solarbio). Serum tumor necrosis factor-alpha (TNF-α) and interleukin-6 (IL-6) were measured using mouse TNF-α ELISA kit (CME0004, 4Abio) and mouse IL-6 ELISA kit (CME0006, 4Abio), respectively.

### Quantitative real-time PCR

RNA from cells and tissues was extracted using the FastPure Cell/Tissue Total RNA Isolation kit (RC101, Vazyme) and reverse transcribed to complementary DNA using the HiScript III RT supermix for qPCR kit (R323, Vazyme). Quantification was performed with ChamQ Universal SYBR qPCR Master Mix (Q711, Vazyme) in a sample reaction system placed on the Roche LightCycler 96 PCR instrument (Roche). The primer sequences are shown in supplemental Table S2.

### Western blot analysis

Cells and tissues were treated with RIPA lysis buffer (IN-WB001, Invent, Eden Prairie) and a total protein extraction kit for animal cultured cells/tissues (SD-001/SN-002, Invent, Eden Prairie, US) to obtain total protein. The extracted proteins were then subjected to electrophoresis on SDS-PAGE gels. For immunoblotting, the following antibody were used: β-actin (1:2000, TA811000, Origene), SDCCAG3 (1:1000, sc-398909), PPARγ (1:1000, A11183, ABclonal), CEBPα (1:1000, sc-166258), SMURF1(1:1000, sc-100616), FALG (1:1000, F1804, Sigma-Aldrich), HA (1:1000, sc-7392), UB (1:2000, PTM-1106RM, PTM BIolabs).

### Cells treatment and induction of differentiation

HEK293T cells were generously provided by RSBM Cell Bank (Taiyuan) and cultured in complete DMEM supplemented with 10% fetal bovine serum (FBS) (S711-001S, Lonsera). 3T3-L1 mouse embryonic fibroblasts were purchased from Shanghai Qida Biotechnology Co., Ltd. and cultured in complete DMEM containing 10% calf serum (80,230-6412, Isite). The adipogenic differentiation induction medium of 3T3-L1 preadipocytes consisted of complete DMEM with 10% FBS, supplemented with 10 μg/ml insulin (HY-P0035, MCE, New Brunswick, US), 1 μM dexamethasone (ID0170, Solarbio) and 0.5 mM 3-isobutyl-1-methylxanthine (IBMX) (HY-12318, MCE), referred to as solution A. Solution B comprised complete DMEM containing 10% FBS and 10 μg/ml insulin. Complete DMEM containing 10% FBS served as solution C. The time of cell confluence was recorded as day 2. After 2 days of confluence, the cocktail strategy was employed: cells were cultured in solution A for 48 h, followed by solution B for another 48 h, and then transitioned to solution C for maintenance, which was changed every other day. The differentiated cells continued to be cultured in either normal medium or high fat medium for 4 days. The DMEM in solution C was replaced with a high-fat medium (AAPR156, PYTHONBIO) for high-fat culture. For transfection, designated plasmids or small interfering RNA (siRNA) were transfected according to the instructions of the Lipo8000 transfection reagent (C0533, Beyotime). Cells were treated with 100 μM Cycloheximide (CHX) (HY-12320, MCE), 20 μM chloroquine (CHQ) (HY-17589A, MCE), and 10 μM MG132 (HY-13259, MCE) for specific durations as required.

### Oil red O and BODIPY staining

Lipid droplet staining was performed using an oil red O stain kit for cultured cells (G1262, Solarbio) according to the manufacturer's instructions. The stained samples were viewed and photographed using an Olympus IX73 microscope (Olympus). For BODIPY staining, cells were first fixed in a 4% paraformaldehyde solution for 30 min and stained with 0.5 μM BODIPY 493/503 (GC42959, GLPBIO) for 30 min, followed by DAPI (C0065, Solarbio) staining for 5 min. The samples were then viewed and photographed using a Leica DMI8 inverted microscope(Leica).

### Co-immunoprecipitation

Cells were harvested using IP lysis buffer (P0013, Beyotime) to obtain total protein. Protein A/G magnetic beads (HY-K0202, MCE) were fully resuspended, and 50 μl was placed in a 1.5 ml EP tube. Subsequently, 400 μl binding/washing buffer (PBST, containing 0.5% Tween-20, pH7.4) was added to wash the beads thoroughly. The 1:50 HA, 1:50 FLAG, 1:200 IgG (B900620, Proteintech) and 1:50 SMURF1 (SC-100616, Santa Cruz, US) antibodies were diluted using binding/washing buffer. 400 μl of diluted antibody solution was added to the magnetic beads and incubated at 4°C for 2 h on a flip mixer. Then the magnetic beads were magnetically separated, collected, and washed thoroughly with binding/washing buffer. Next, 400 μl of the cell protein sample was added into the magnetic beads and incubated at 4°C for 2 h. The beads were magnetically separated, and the supernatant was discarded. After thorough washing, 50 μl of 1×SDS-PAGE loading buffer (P0015 L, Beyotime) was added to the beads, mixed well, and heated at 95°C for 5 min. The magnetic beads were separated to collect the supernatant, which was subsequently subjected to SDS-PAGE analysis.

### Dual luciferase reporter gene assay

HEK293T cells were seeded in 24-well plates. The mouse *Pparγ* luciferase reporter gene plasmid, the renilla luciferase gene plasmid and either the mouse *Sdccag3* or control plasmid were co-transfected. Mouse *Sdccag3* luciferase reporter gene plasmid or *Sdccag3* mutant luciferase reporter gene plasmid, along with the renilla luciferase gene plasmid and either the mouse *Pparγ* or control plasmid were co-transfected. After 24 h of transfection, the cells were collected and treated with a dual luciferase reporter assay kit (DL101-01, Vazyme). The luciferase activity was then detected by Synergy H1 microplate reader (Biotek).

### Data statistics and analysis

All in vitro experiments were conducted with three independent replicates. Animal experiments were performed using random grouping, and all sample sizes (n-values) were indicated in the figure legends. All data were analyzed using GraphPad Prism 9.0 software. Comparisons between the two groups were statistically analyzed by two-tailed *t* test. For multiple comparisons with two independent variables, two-way ANOVA was used. Data were presented as mean ± standard deviation (S.D.). *P*-values <0.05 were considered as statistically significant.

## Results

### Determining SDCCAG3 as a regulator of adipocyte metabolism

**Fig. 1:**
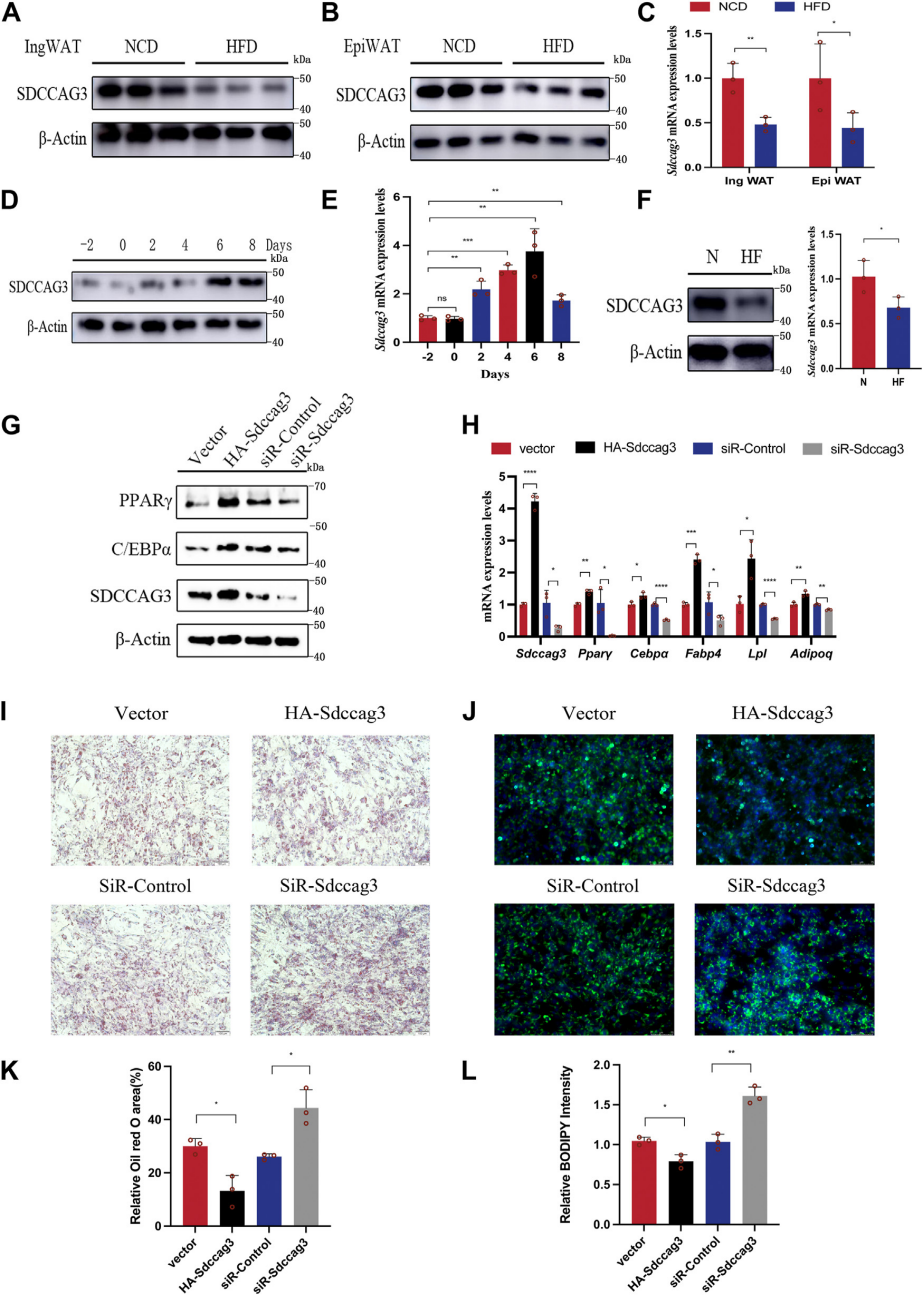
Determining SDCCAG3 as a regulator of adipocyte metabolism. A: Protein expression levels of SDCCAG3 in IngWAT of mice on NCD or HFD. B: Protein expression levels of SDCCAG3 in EpiWAT of mice on NCD or HFD. C: SDCCAG3 mRNA levels in IngWAT and EpiWAT of mice on NCD or HFD. D and E: Changes in SDCCAG3 protein and mRNA levels during the differentiation of 3T3-L1 cells into mature adipocytes. F: Protein and mRNA levels of SDCCAG3 in differentiated 3T3-L1 cells cultured under normal or high-fat conditions. G: Protein expression levels of PPARγ, C/EBPα and SDCCAG3 with knockdown or overexpression of SDCCAG3 on differentiated 3T3-L1 cells cultured under high-fat conditions. H: SDCCAG3, PPARγ, C/EBPα, FABP4, LPL and ADIPOQ mRNA levels with knockdown or overexpression of SDCCAG3 on differentiated 3T3-L1 cells cultured under high-fat conditions. I and K: Oil red O staining of differentiated 3T3-L1 cells and relative area with knockdown or overexpression of SDCCAG3, scale bar = 100 μm. J and L: BODIPY staining of differentiated 3T3-L1 cells and relative intensity with knockdown or overexpression of SDCCAG3, scale bar = 75 μm. Data were presented as mean ± standard deviation (S.D.). ∗*P* < 0.05, ∗∗*P* < 0.01, ∗∗∗*P* < 0.001, ∗∗∗∗*P* < 0.0001.

**Supplementary Figure S1:**
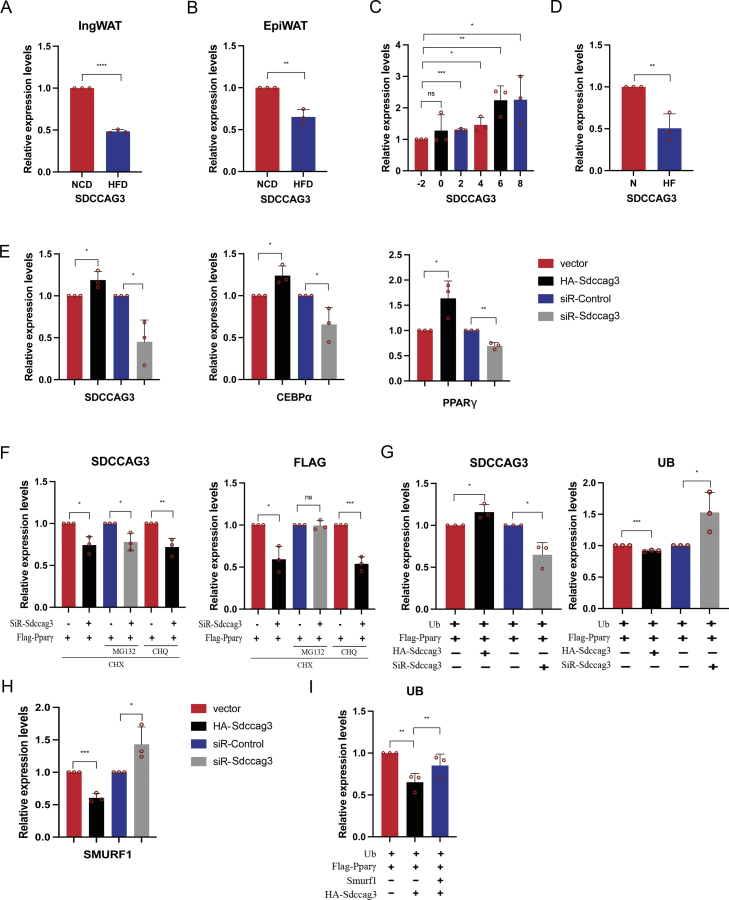


Our previous research found that the HFD altered the expression levels of SDCCAG3 in the bone tissue of mice (28). To investigate the expression changes of SDCCAG3 in the adipose tissue of obese mice induced by HFD, we compared the expression levels of SDCCAG3 in the adipose tissue mice fed NCD versus those on HFD for 16 weeks. The results showed that, compared to mice on NCD, both the gene and protein expression levels of SDCCAG3 in the epididymal white adipose tissue (EpiWAT) and inguinal white adipose tissue (IngWAT) of HFD mice were significantly reduced (Fig. 1A–C and supplemental Fig. S1A, B). Therefore, we hypothesized that SDCCAG3 may exert a potential impact on adipose tissue.

During the differentiation of 3T3-L1 preadipocytes into mature adipocytes, SDCCAG3 was stably induced (Fig. 1D, E and supplemental Fig. S1C). Subsequently, we continued to culture the differentiated adipocytes in either normal or high-fat media. Immunoblotting and qPCR analyses revealed that the gene and protein expression levels of SDCCAG3 were decreased in adipocytes under high-fat conditions (Fig. 1F and supplemental Fig. S1D). To further investigate the role of SDCCAG3 in adipocytes, we confirmed its effects in a high-fat environment by transfecting SDCCAG3 plasmids and siRNA. Immunoblotting and qPCR analyses indicated that the overexpression of SDCCAG3 led to an upregulation of mRNA and protein levels of PPARγ and CCAAT/enhancer-binding protein alpha (C/EBP), as well as an increase in the mRNA levels of fatty acid binding protein 4 (FABP4), lipoprotein lipase (LPL) and adiponectin (ADIPOQ) (Fig. 1G, H and supplemental Fig. S1E). Results from Oil Red O and BODIPY staining demonstrated that the upregulation of SDCCAG3 in adipocytes reduced lipid accumulation under high-fat conditions compared to the control group (Fig. 1I–L). Conversely, knocking down SDCCAG3 resulted in decreased expression levels of PPARγ, C/EBPα, FABP4, LPL and ADIPOQ in adipocytes under high-fat conditions, while lipid accumulation increased. These findings were consistent with the characteristics of adipocyte hypertrophy and downregulation of lipid homeostasis regulatory factors induced by high-fat conditions. This indicated that SDCCAG3 played a critical role in the metabolic regulation of adipocytes.

### SDCCAG3 was implicated in the regulation of PPARγ protein stability

**Fig. 2:**
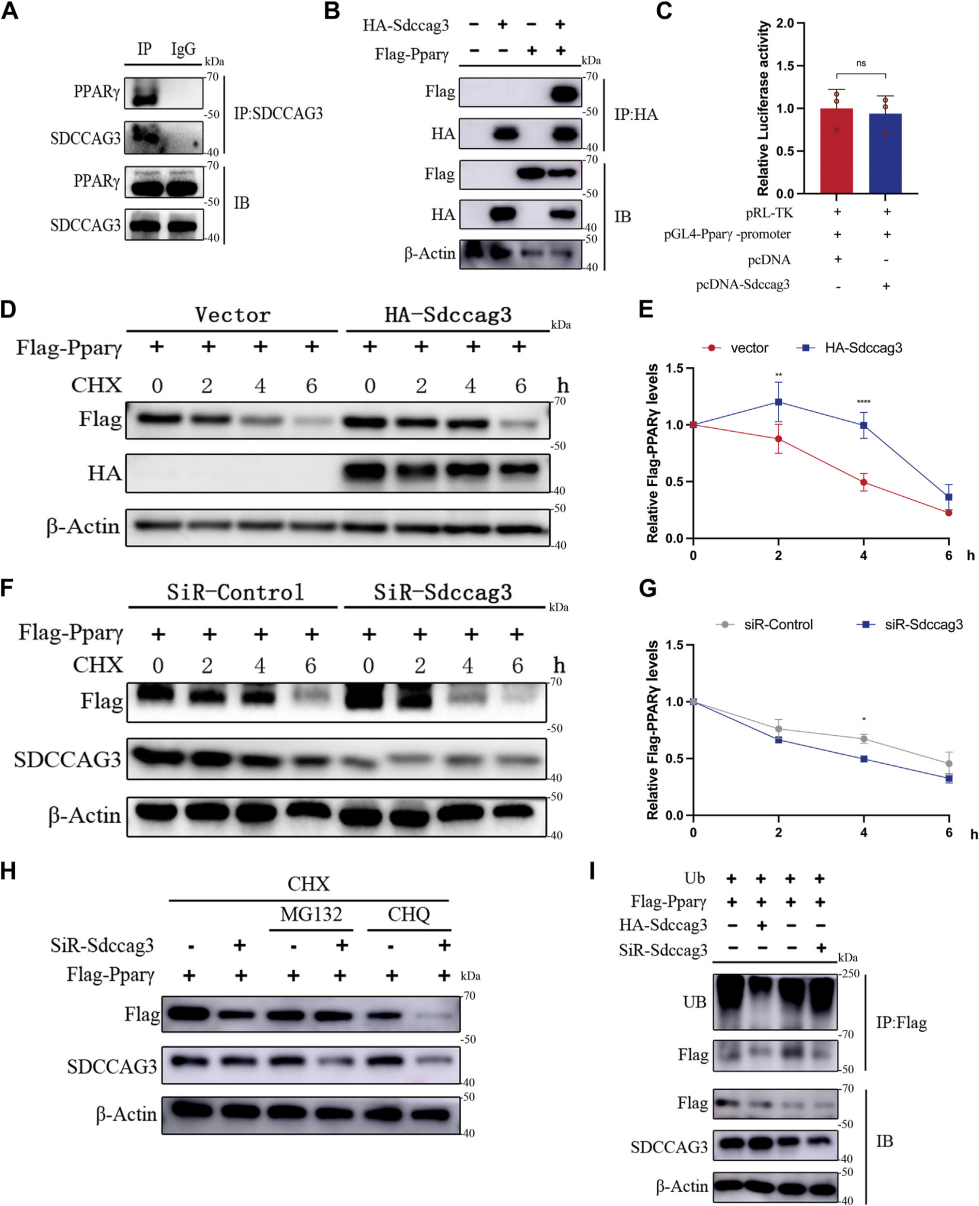
SDCCAG3 promoted the protein stability of PPARγ. A: Co-immunoprecipitation of SDCCAG3 and PPARγ in 3T3-L1 adipocytes was detected using SDCCAG3 antibody binding. B: Co-immunoprecipitation of SDCCAG3 and PPARγ in HEK293T cells exogenously expressing HA-Sdccag3 and Flag-Pparγ. C: Luciferase activity was measured following the transfection of HEK293T cells with PPARγ luciferase reporter gene plasmid, renilla luciferase gene plasmid and SDCCAG3 plasmid. D and E: Protein expression levels of Flag-PPARγ and HA-SDCCAG3 in HEK293T cells overexpressing HA-Sdccag3 and Flag-Pparγ after treated with CHX for 0, 2, 4 and 6h. F and G: Protein expression levels of Flag-PPARγ and SDCCAG3 in HEK293T cells of knockdown SDCCAG3 and overexpression Flag-Pparγ after treated with CHX for 0, 2, 4 and 6h. H: In HEK293T cells with SDCCAG3 knockdown, Flag-PPARγ and SDCCAG3 protein expression levels were detected after treatment with CHX or dual treatment with MG132 and CHQ for 4h. I: After SDCCAG3 knockdown or overexpression in HEK293T cells treated with UB and Flag-Pparγ plasmids, Flag-PPARγ protein was pulled down by magnetic beads, and ubiquitination levels were detected by Western blot. Data were presented as mean ± standard deviation (S.D.). ∗*P* < 0.05, ∗∗*P* < 0.01, ∗∗∗*P* < 0.001, ∗∗∗∗*P* < 0.0001.

Our previous research has demonstrated that SDCCAG3 can regulate the expression of PPARγ in bone marrow mesenchymal stem cells. Subsequently, we investigated the potential interaction between SDCCAG3 and PPARγ in adipocytes. Co-immunoprecipitation analysis confirmed the interaction between SDCCAG3 and endogenous PPARγ in adipocytes (Fig. 2A). Additionally, in HEK293T cells, HA-tagged SDCCAG3 was shown to interact with exogenously expressed FLAG-tagged PPARγ (Fig. 2B).

To elucidate the mechanism of action of SDCCAG3, we first conducted a dual-luciferase reporter assay, which demonstrated that SDCCAG3 did not activate the PPARγ promoter (Fig. 2C). This finding indicated that SDCCAG3 was unable to directly activate the PPARγ promoter to regulate its expression at the transcriptional level. Subsequently, we observed that the degradation rate of PPARγ protein was significantly reduced when FLAG-tagged PPARγ and HA-tagged SDCCAG3 were co-expressed, particularly when protein translation was inhibited using CHX (Fig. 2D, E). Conversely, knockdown of SDCCAG3 markedly accelerated the degradation rate of PPARγ protein (Fig. 2F, G). Furthermore, in the presence of the proteasome inhibitor MG132, the degradation of PPARγ protein induced by the knockdown of SDCCAG3 was inhibited. In contrast, the degradation of PPARγ protein resulting from SDCCAG3 knockdown was not affected by the lysosome inhibitor CHQ (Fig. 2H and supplemental Fig. S1F). These results indicated that SDCCAG3 played a regulatory role in the PPARγ-dependent proteasomal degradation pathway.

Given that C-terminal ubiquitination of PPARγ leads to its proteasomal degradation, we investigated the influence of SDCCAG3 on this process. As anticipated, overexpression of SDCCAG3 reduced PPARγ ubiquitination, whereas knockdown of SDCCAG3 enhanced PPARγ ubiquitination (Fig. 2I and supplemental Fig. S1G). These alterations in PPARγ ubiquitination levels may subsequently affect its protein expression through the proteasomal degradation pathway.

### SMURF1 was involved in the regulation of PPARγ protein stability by interacting with SDCCAG3

**Fig. 3:**
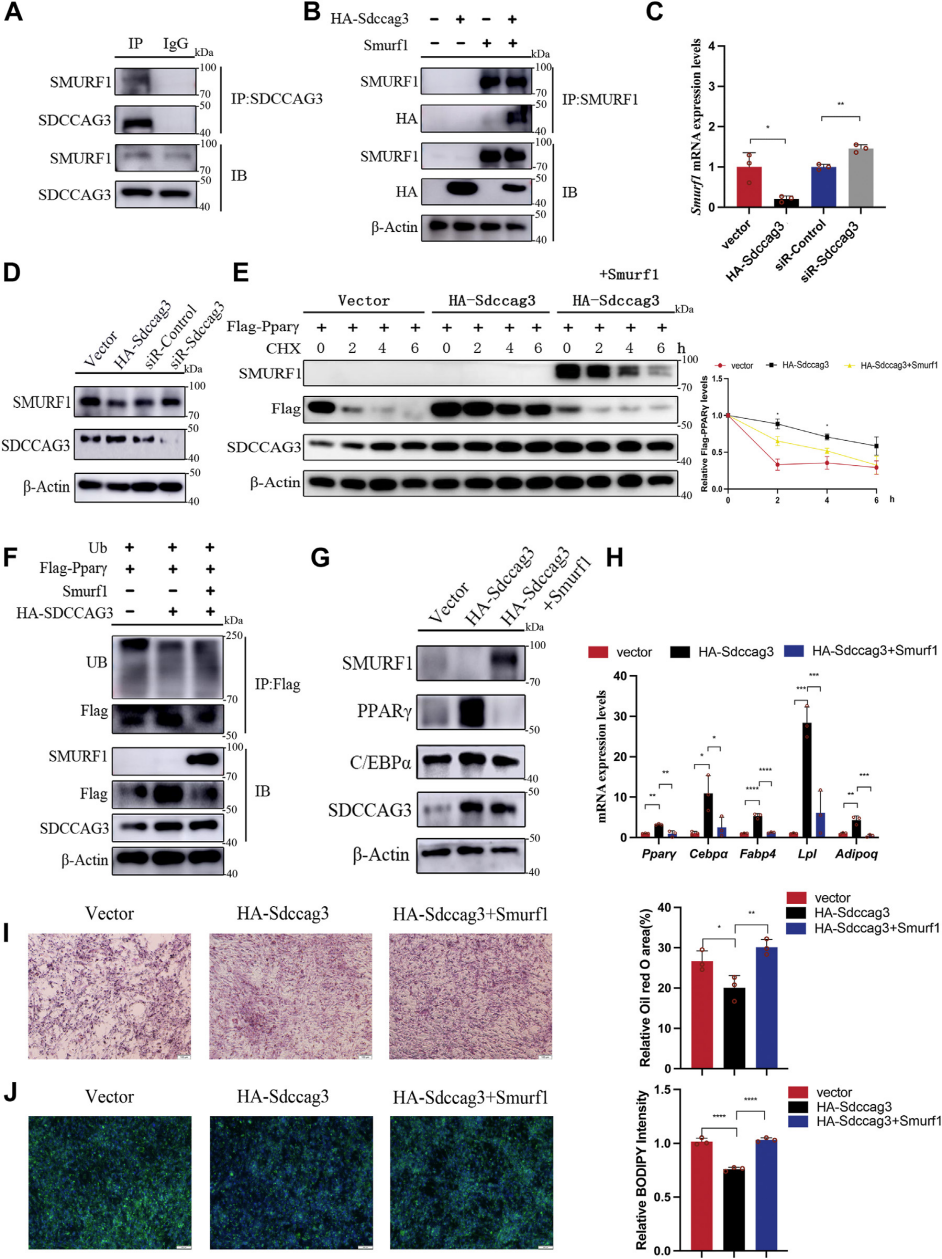
SMURF1 mediated the regulation of SDCCAG3 on PPARγ protein stability and adipocyte. A: Co-immunoprecipitation of SDCCAG3 and SMURF1 in 3T3-L1 cells. B: Co-immunoprecipitation of SDCCAG3 and SMURF1 in HEK293T cells exogenously expressing HA-Sdccag3 and Smurf1. C: The mRNA levels of SMURF1 with knockdown or overexpression of SDCCAG3 on differentiated 3T3-L1 cells. D: Protein expression levels of SMURF1 and SDCCAG3 with knockdown or overexpression of SDCCAG3 on differentiated 3T3-L1 cells. E: The HEK293T cells overexpressing Flag-Pparγ were co-transfected with HA-Sdccag3 or Smurf1, and the protein expression levels of Flag-PPARγ, SMURF1 and SDCCAG3 were detected after CHX treatment for 0, 2, 4, and 6h. F: HA-Sdccag3 or Smurf1 were co-transfected with UB and Flag-Pparγ plasmids into HEK293T cells. Flag-PPARγ protein was pulled down by magnetic beads, and the ubiquitination levels were detected by Western blot. G: Protein expression levels of PPARγ, C/EBPα, SDCCAG3 and SMURF1 with co-transfection of Smurf1 and HA-Sdccag3 on differentiated 3T3-L1 cells cultured under high-fat conditions. H: PPARγ, C/EBPα, FABP4, LPL and ADIPOQ mRNA levels with co-transfection of Smurf1 and HA-Sdccag3 on differentiated 3T3-L1 cells cultured under high-fat conditions. I: Oil red O staining and relative area of differentiated 3T3-L1 cells cultured under high-fat conditions with co-transfection of Smurf1 and HA-Sdccag3, scale bar = 100 μm. J: BODIPY staining and relative intensity of differentiated 3T3-L1 cells cultured under high-fat conditions with co-transfection of Smurf1 and HA-Sdccag3, scale bar = 200 μm. Data were presented as mean ± standard deviation (S.D.). ∗*P* < 0.05, ∗∗*P* < 0.01, ∗∗∗*P* < 0.001, ∗∗∗∗*P* < 0.0001.

**Supplementary Figure S2:**
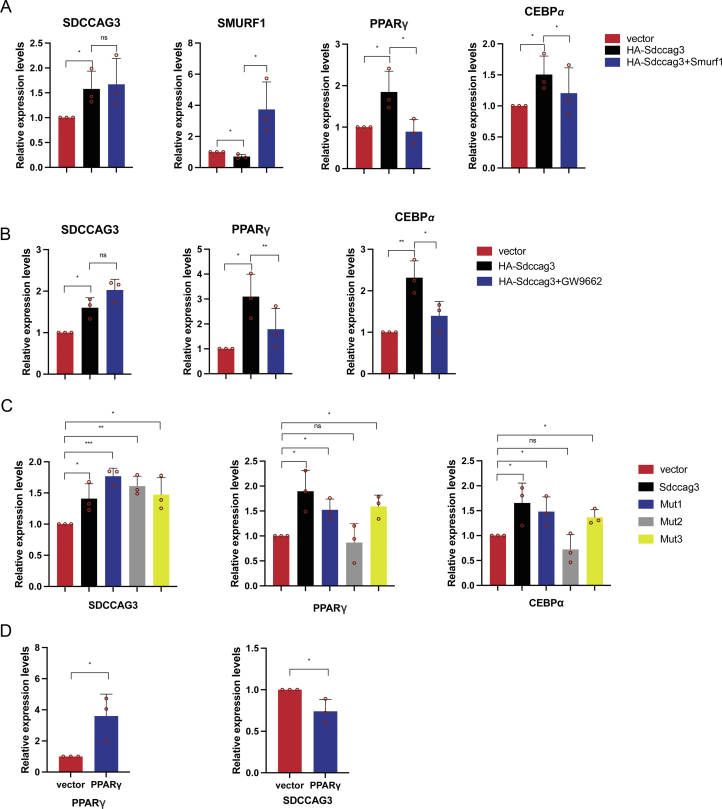


SDCCAG3 influenced the ubiquitination of PPARγ, thereby impacting its protein stability. However, some studies have reported that SDCCAG3 lacks enzymatic activity (25, 26). Consequently, we postulated the existence of a regulatory factor that governs PPARγ stability and is itself regulated by SDCCAG3. In our previous research, we identified interactions between SDCCAG3 and several proteins, including the ubiquitin ligase SMURF1, which was known to directly ubiquitinate PPARγ. SMURF1 promoted PPARγ degradation through this ubiquitination process (30). Subsequent co-immunoprecipitation analysis confirmed the interaction of SMURF1 and endogenous SDCCAG3 in adipocytes (Fig. 3A). Additionally, SMURF1 was observed to interact with exogenously expressed HA-tagged SDCCAG3 (Fig. 3B). Immunoblotting and qPCR analyses demonstrated that overexpression of SDCCAG3 reduced expression of SMURF1 in adipocytes, while knockdown of SDCCAG3 led to an increase in SMURF1 expression (Fig. 3C, D and supplemental Fig. S1H). Treatment with CHX revealed that SMURF1 can counteract the enhancing effect of SDCCAG3 on PPARγ protein stability and reverse the ubiquitination of PPARγ (Fig. 3E, F and supplemental Fig. S1I). Moreover, in adipocytes overexpressing SDCCAG3, simultaneous overexpression of SMURF1 revealed that SMURF1 could inhibit the upregulation of lipid metabolism-related factors and the reduction of lipid accumulation induced by SDCCAG3 in a high-fat environment (Fig. 3G–J and supplemental Fig. S2A). Therefore, these findings supported our hypothesis that SMURF1 serves as an intermediate regulatory factor in the regulation of PPARγ stability by SDCCAG3 and is itself regulated by SDCCAG3.

### SDCCAG3 played a regulatory role in adipocytes in a PPARγ-dependent manner

**Fig. 4:**
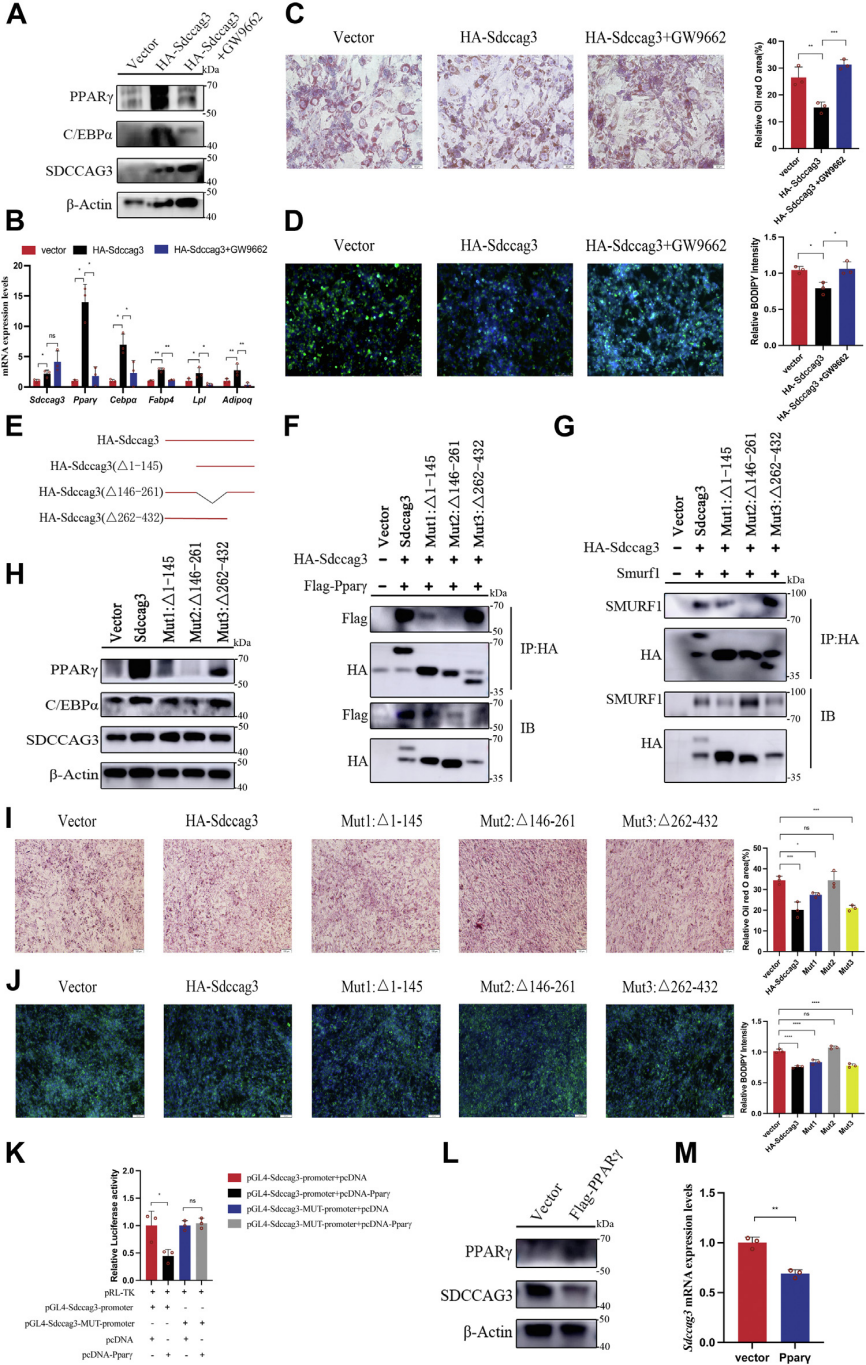
Determining the functional fragment of SDCCAG3 and a SDCCAG3-PPARγ-SDCCAG3 lipid metabolism enhancement loop. A: Protein expression levels of SDCCAG3, PPARγ, C/EBPα in differentiated 3T3-L1 cells transfected with HA-Sdccag3 and treated with GW9662 under high-fat conditions. B: The mRNA levels of SDCCAG3, PPARγ, C/EBPα, FABP4, LPL and ADIPOQ in differentiated 3T3-L1 cells transfected with HA-Sdccag3 and treated with GW9662 under high-fat conditions. C: Differentiated 3T3-L1 cells transfected with HA-Sdccag3 were treated with GW9662 and stained with oil red O, scale bar = 50 μm. D: Differentiated 3T3-L1 cells transfected with HA-Sdccag3 were treated with GW9662 and stained with BODIPY, scale bar = 75 μm. E: Example plots of full-length Sdccag3 and its mutants lacking segments 1–145, 146–261, and 262–432, respectively. F: In HEK293T cells, full-length Sdccag3 and its mutants (mut1, mut2, and mut3) were transfected with Flag-Pparγ, and the co-immunoprecipitation of HA and Flag was detected. G: In HEK293T cells, full-length Sdccag3 and its mutants (mut1, mut2, and mut3) were transfected with Smurf1, and the co-immunoprecipitation of HA and SMURF1 was detected. H: Protein expression levels of PPARγ, C/EBPα and SDCCAG3 with transfection of full-length Sdccag3 and its mutants (mut1, mut2, and mut3) on differentiated 3T3-L1 cells. I: Oil red O staining and relative area of differentiated 3T3-L1 cells with transfection of full-length Sdccag3 and its mutants (mut1, mut2, and mut3), scale bar = 100 μm. J: BODIPY staining and relative intensity of differentiated 3T3-L1 cells with transfection of full-length Sdccag3 and its mutants (mut1, mut2, and mut3), scale bar = 200 μm. K: Luciferase activity was measured after transfection of Sdccag3 or mutant luciferase reporter gene plasmid, renilla luciferase gene plasmid and Pparγ plasmid in HEK293T cells. L: Protein expression levels of PPARγ and SDCCAG3 in differentiated 3T3-L1 cells overexpressing Flag-Pparγ. M: SDCCAG3 mRNA levels in differentiated 3T3-L1 cells overexpressing Flag-Pparγ. Data were presented as mean ± standard deviation (S.D.). ∗*P* < 0.05, ∗∗*P* < 0.01, ∗∗∗*P* < 0.001, ∗∗∗∗*P* < 0.0001.

We further explored whether the regulatory effect of SDCCAG3 in adipocytes is contingent upon its regulation of PPARγ. GW9662, a selective PPARγ antagonist, was employed for this purpose. As evidenced by Immunoblotting and qPCR, GW9662 significantly reversed the upregulation of lipid metabolism-related factors induced by SDCCAG3 overexpression in adipocytes under high-fat conditions (Fig. 4A, B and supplemental Fig. S2B). Additionally, GW9662 effectively reversed the reduction in lipid accumulation, as demonstrated by Oil Red O and BODIPY staining (Fig. 4C, D). These findings confirmed that the regulatory effect of SDCCAG3 in adipocytes was at least partially dependent on the function of PPARγ.

### Identifying the specific functional domains of SDCCAG3

In our quest to identify the specific functional segments of SDCCAG3, we partitioned the protein into three distinct domains based on its structural architecture (supplemental Tables S3–S6). Subsequently, we constructed plasmids containing HA-tagged fragment deletion mutants corresponding to these domains (Fig. 4E). Co-immunoprecipitation analysis revealed that the SDCCAG3 mutant lacking the intermediate segment (encompassing the validated PTPN13-interacting region (26), polar residues region, and disordered region) failed to interact with PPARγ (Fig. 4F). Furthermore, no interaction with SMURF1 was detected under similar conditions (Fig. 4G). Subsequently, we proceeded to validate the functional roles of each variant in adipocytes. As anticipated, the aforementioned SDCCAG3 mutants exhibited no regulatory effect on lipid accumulation or on lipid metabolism-related factors in adipocytes under high-fat conditions (Fig. 4H–J and supplemental Fig. S2C). These data confirmed that the specific functional fragment of SDCCAG3 is located between amino acid 146 and 261 in the central region. This discovery provided a theoretical foundation for our future translational applications.

### PPARγ negatively regulated SDCCAG3, forming a SDCCAG3-PPARγ-SDCCAG3 lipid metabolism enhancement loop

Prior research has revealed differential expression of SDCCAG3 under distinct lipid levels contexts. To ascertain the upstream regulatory elements governing SDCCAG3, we utilized the Jaspar database (https://jaspar.genereg.net) to predict transcription factor binding sites within the SDCCAG3 gene. Notably, we found an association between the transcription factor PPARγ and the promoter region of SDCCAG3, suggesting a potential role for PPARγ in modulating the transcriptional activity of SDCCAG3 (supplemental Table S7). Subsequent experimental validation using dual luciferase reporter gene assays confirmed that PPARγ can attenuate the transcriptional activity of SDCCAG3. Moreover, mutant variants lacking specific binding sequences within the SDCCAG3 promoter exhibited no suppression of transcriptional activity (Fig. 4K). Furthermore, overexpression of PPARγ in adipocytes resulted in a reduction of SDCCAG3 expression levels (Fig. 4L, M and supplemental Fig. S2D). These results collectively indicated that PPARγ functioned as a negative feedback regulator controlling the expression level of SDCCAG3, thereby establishing a regulatory loop involving SDCCAG3-PPARγ-SDCCAG3 in lipid metabolism processes.

### Adipose-specific knockout of SDCCAG3 aggravated adipocyte hypertrophy and regulated the expression of lipid metabolic gene programs

In order to investigate the specific role of SDCCAG3 deficiency in adipose tissue function, we utilized the FABP4-Cre-loxP system to generate adipose-specific SDCCAG3 knockout mice. We crossed *Sdccag3*^*FLOX/+*^ mice, which carried loxP sites, with FABP4-cre knock-in mice to produce *Sdccag3*^*FLOX/FLOX*^; FABP4-Cre mice (AKO-*Sdccag3*). *Sdccag3*^*FLOX/FLOX*^ mice that did not express the FABP4-Cre enzyme served as the control group (AKO-WT). The genotype was determined through agarose gel electrophoresis (supplemental Table S1). The feeding regimen of HFD or NCD was initiated at 6 weeks of age and continued for 16 weeks.

**Fig. 5:**
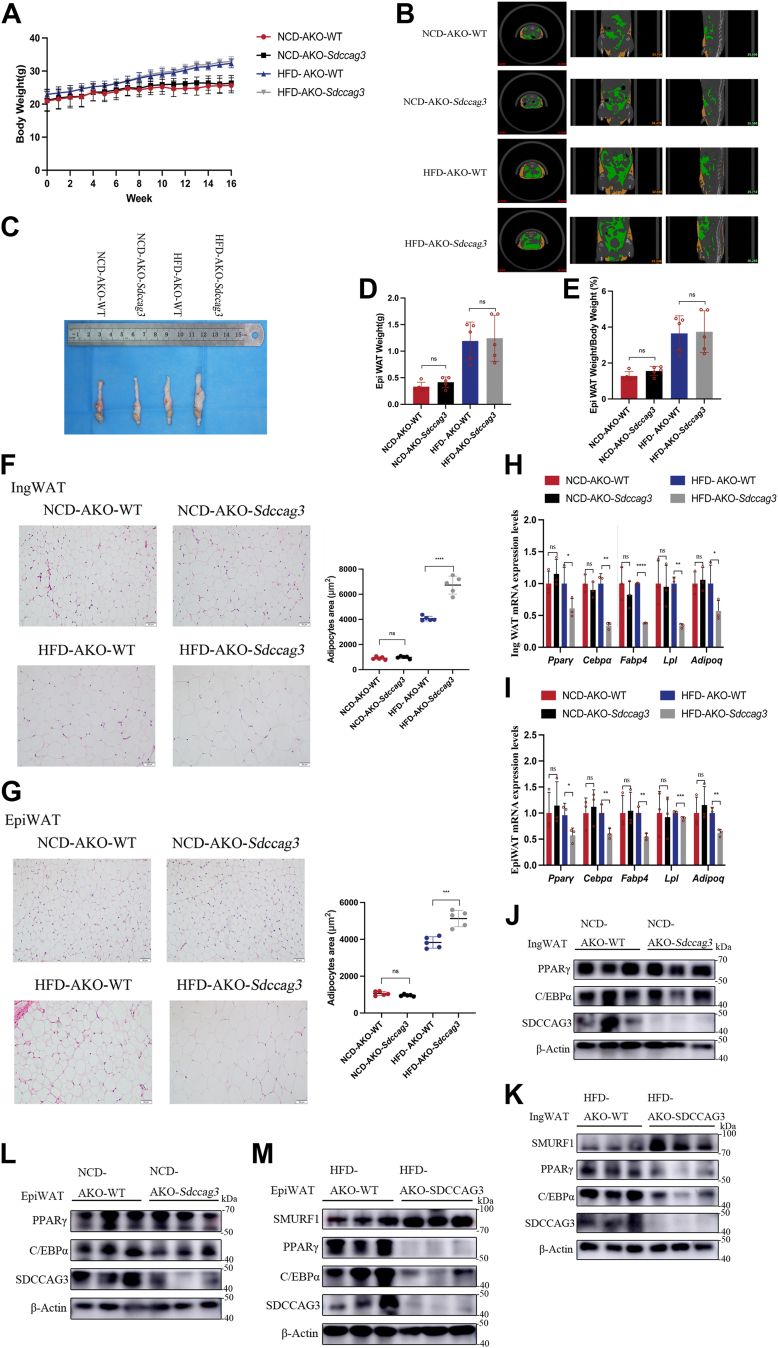
Adipose-specific knockout of SDCCAG3 aggravated adipocyte hypertrophy and abnormal lipid-related indexes. A: Body weight changes in AKO-WT and AKO-*Sdccag3* groups over the course of 16 weeks of NCD or HFD feeding, n = 5. B: Micro-CT scans of AKO-WT and AKO-*Sdccag3* groups of NCD or HFD feeding to reconstruct subcutaneous fat and visceral fat maps. Orange, subcutaneous fat; Green, visceral fat. C: Photos of EpiWAT from AKO-WT and AKO-*Sdccag3* groups of NCD or HFD feeding. D: EpiWAT weight of NCD or HFD-fed AKO-WT and AKO-*Sdccag3* groups, n = 5. E: Ratio of EpiWAT weight to body weight in NCD or HFD-fed AKO-WT and AKO-*Sdccag3* groups, n = 5. F: HE staining of IngWAT and mean area of adipocytes from NCD or HFD-fed AKO-WT and AKO-*Sdccag3* groups, scale bar = 50 μm. G: HE staining of EpiWAT and mean area of adipocytes from NCD or HFD-fed AKO-WT and AKO-*Sdccag3* groups, scale bar = 50 μm. H and I: PPARγ, C/EBPα, FABP4, LPL and ADIPOQ mRNA levels in IngWAT and EpiWAT of NCD or HFD-fed AKO-WT and AKO-*Sdccag3* groups. J: Protein expression levels of PPARγ, C/EBPα and SDCCAG3 in IngWAT of NCD-fed AKO-WT and AKO-*Sdccag3* groups. K: Protein expression levels of PPARγ, C/EBPα, SDCCAG3 and SMURF1 in IngWAT of HFD-fed AKO-WT and AKO-*Sdccag3* groups. L: Protein expression levels of PPARγ, C/EBPα and SDCCAG3 in EpiWAT of NCD-fed AKO-WT and AKO-*Sdccag3* groups. M: Protein expression levels of PPARγ, C/EBPα, SDCCAG3 and SMURF1 in EpiWAT of HFD-fed AKO-WT and AKO-*Sdccag3* groups. Data were presented as mean ± standard deviation (S.D.). ∗*P* < 0.05, ∗∗*P* < 0.01, ∗∗∗*P* < 0.001, ∗∗∗∗*P* < 0.0001.

**Supplementary Figure S3:**
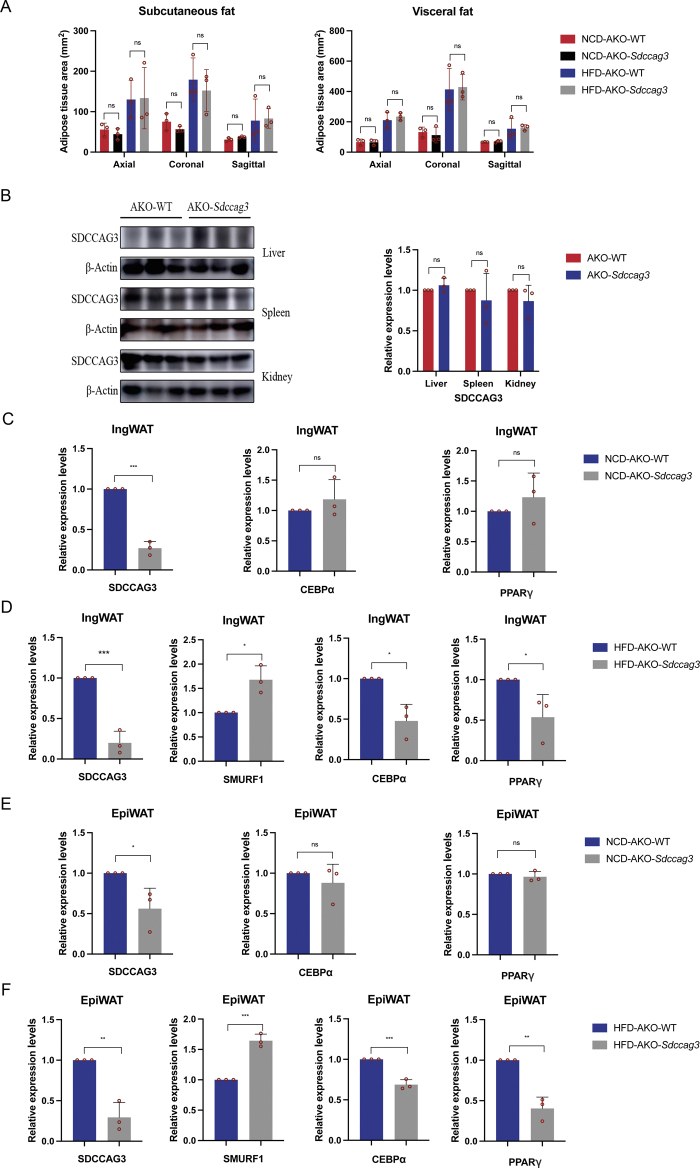


We measured body weight weekly and found a noticeable difference in body weight between the NCD and HFD groups by the eighth week. However, within each dietary condition, the two groups of mice did not show significant differences in body weight (Fig. 5A). To further assess the impact of adipose-specific SDCCAG3 knockout on adipose tissue, we conducted Micro-CT analysis to examine abdominal adipose tissue. The Micro-CT analysis demonstrated that the adipose mass of AKO-*Sdccag3* was comparable to that of AKO-WT in both the NCD and HFD feeding groups (Fig. 5B and supplemental Fig. S3A). Subsequently, we weighed the excised EpiWAT from the mice, and the analysis revealed that the volume of EpiWAT in AKO-*Sdccag3* was similar to that in AKO-WT across both NCD and HFD feeding groups, with no significant differences observed in EpiWAT weight or the EpiWAT weight/body weight ratio (Fig. 5C–E). These findings suggested that the adipose-specific knockout of SDCCAG3 did not affect weight gain or the increase in adipose tissue weight associated with HFD.

Next, to evaluate the effect of adipose-specific SDCCAG3 knockout on adipocyte size, we performed histological analysis of IngWAT and EpiWAT using HE staining. We found that, compared to the HFD-AKO-WT group, adipocyte size of the HFD-AKO-*Sdccag3* mice was increased, while no significant changes were observed in the NCD feeding groups. Quantitative analysis corroborated these findings (Fig. 5F, G). These results suggestd that SDCCAG3 in adipose tissue played a specific regulatory role in the size of adipocyte under high-fat dietary conditions.

Adipocyte hypertrophy is an important feature leading to adipose tissue dysfunction and metabolic disorders. We examined the gene expression levels of metabolic regulatory factors involved in adipose tissue homeostasis, including PPARγ, C/EBPα, FABP4, LPL and ADIPOQ. Compared to the HFD-AKO-WT control group, the expression levels of these genes in the IngWAT and EpiWAT of HFD-AKO-*Sdccag3* mice were significantly reduced, while no significant changes were observed in the NCD feeding groups (Fig. 5H, I). These findings align with the characteristics of metabolically impaired hypertrophic adipocytes. In the previous description, we mentioned that genotyping had been performed. Additionally, Western blot analysis revealed a significant reduction in the expression of SDCCAG3 in the IngWAT and EpiWAT of AKO-*Sdccag3* mice compared to AKO-WT mice. This reduction was not observed in other tissues, further confirming the specific knockout of SDCCAG3 in adipose tissue (supplemental Fig. S3B). Dysfunction of PPARγ is a hallmark of hypertrophic obesity. In the IngWAT and EpiWAT of HFD-AKO-*Sdccag3* mice, we observed a significant downregulation of PPARγ and its target protein C/EBPα, while the expression levels of lipid-related factors remained unaffected in the NCD feeding groups (Fig. 5J–M and supplemental Fig. S3C–F). These data indicated that adipose-specific knockout of SDCCAG3 exacerbated adipocyte hypertrophy under high-fat dietary conditions and downregulated the expression levels of lipid metabolism genes, potentially worsening dysfunction and metabolic disturbances in adipose tissue.

### Adipose-specific knockout of SDCCAG3 impaired glucose tolerance and increased insulin resistance

**Fig. 6:**
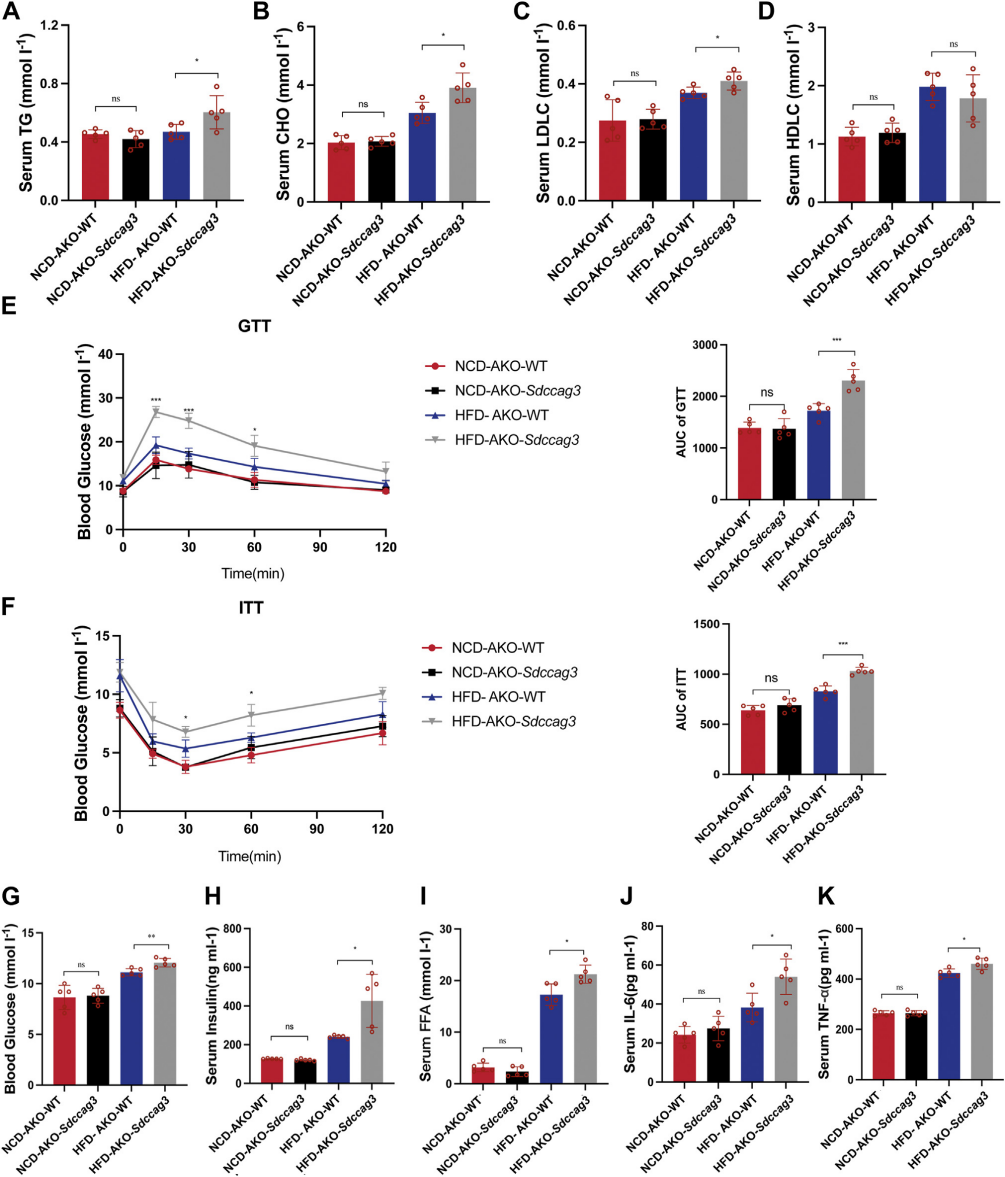
Adipose-specific knockout of SDCCAG3 aggravated insulin resistance and obesity-related metabolic disorders. A: Serum triglyceride levels in NCD or HFD-fed AKO-WT and AKO-*Sdccag3* groups, n = 5. B: Serum cholesterol levels in NCD or HFD-fed AKO-WT and AKO-*Sdccag3* groups, n = 5. C: Serum low density lipoprotein cholesterol levels in NCD or HFD-fed AKO-WT and AKO-*Sdccag3* groups, n = 5. D: Serum high density lipoprotein cholesterol levels in NCD or HFD-fed AKO-WT and AKO-*Sdccag3* groups, n = 5. E: Glucose tolerance test and area under curve in NCD or HFD-fed AKO-WT and AKO-*Sdccag3* groups, n = 5. F: Insulin tolerance test and area under curve in NCD or HFD-fed AKO-WT and AKO-*Sdccag3* groups, n = 5. G: Fasting serum glucose levels in NCD or HFD-fed AKO-WT and AKO-*Sdccag3* groups, n = 5. H: Fasting serum insulin levels in NCD or HFD-fed AKO-WT and AKO-*Sdccag3* groups, n = 5. I: Serum free fatty acid levels in NCD or HFD-fed AKO-WT and AKO-*Sdccag3* groups, n = 5. J: Serum IL-6 levels in NCD or HFD-fed AKO-WT and AKO-*Sdccag3* groups, n = 5. K: Serum TNF-α levels in NCD or HFD-fed AKO-WT and AKO-*Sdccag3* groups, n = 5. Data were presented as mean ± standard deviation (S.D.). ∗*P* < 0.05, ∗∗*P* < 0.01, ∗∗∗*P* < 0.001, ∗∗∗∗*P* < 0.0001.

Given the close association between obesity and type 2 diabetes, we subsequently assessed the lipid levels in the serum and the glucose homeostasis of the mice. In the NCD groups, no significant changes were observed in the levels of TG, CHO, LDLC and HDLC. Conversely, HFD-AKO-*Sdccag3* mice exhibited elevated levels of TG, CHO and LDLC compared to HFD-AKO-WT mice, while HDLC levels showed no significant difference (Fig. 6A–D). Next the glucose tolerance test indicated that HFD-AKO-*Sdccag3* mice had reduced blood glucose regulation and exhibited impaired glucose tolerance (Fig. 6E). Meanwhile, the insulin tolerance test showed that HFD-AKO-*Sdccag3* mice demonstrated resistance to the glucose-lowering effects of insulin. In the NCD groups, there was minimal difference in the blood glucose change curves (Fig. 6F). We also found that the levels of blood glucose, insulin and FFA in HFD-AKO-*Sdccag3* mice were higher than those in HFD-AKO-WT mice. In contrast to the results observed in the HFD groups, the absence of SDCCAG3 had no impact on these indicators in the NCD groups (Fig. 6G–I). Additionally, there were no significant differences in the serum inflammatory factors TNF-α and IL-6 between the NCD groups, while these levels were elevated in HFD-AKO-*Sdccag3* mice compared to HFD-AKO-WT mice (Fig. 6J, K). These data suggested that the deficiency of SDCCAG3 exacerbated glucose tolerance impairment and insulin resistance under high-fat dietary conditions, as well as promoted the rise of inflammation-related indicators and serum lipid levels.

**Supplementary Figure S4:**
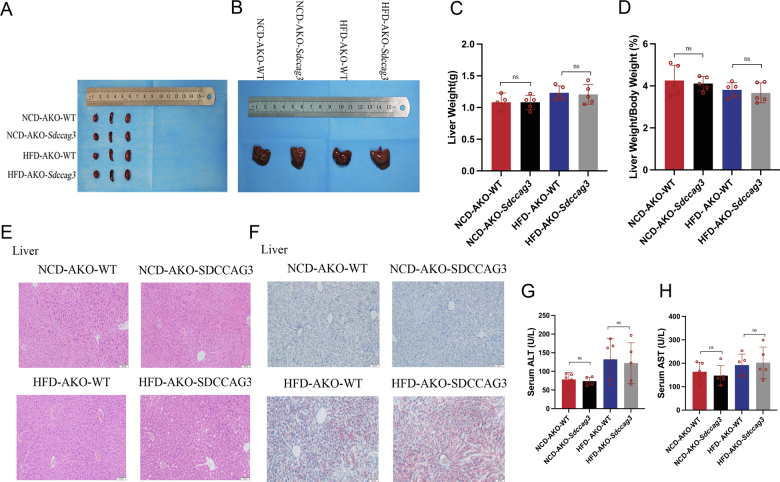


We also examined the morphology of the liver, heart, spleen, and kidneys. No significant changes were observed in the size of these organs (supplemental Fig. S4A, B). Furthermore, there were no differences in liver weight and the liver weight/body weight ratio between the NCD and HFD groups (supplemental Fig. S4C, D). Since the liver is an important organ for lipid metabolism, HE and Oil Red O staining indicated that HFD-AKO-*Sdccag3* mice exhibited increased lipid accumulation in the liver (supplemental Fig. S4E, F). Additionally, no differences were observed in serum liver function markers ALT and AST between the NCD and HFD groups (supplemental Fig. S4G, H). These findings suggested that the liver was not entirely spared from the lipid metabolism disorders caused by SDCCAG3 knockout under systemic conditions.

### Adipose-specific overexpression of SDCCAG3 reduced adipocyte size and promoted the expression of lipid metabolic gene programs

**Fig. 7:**
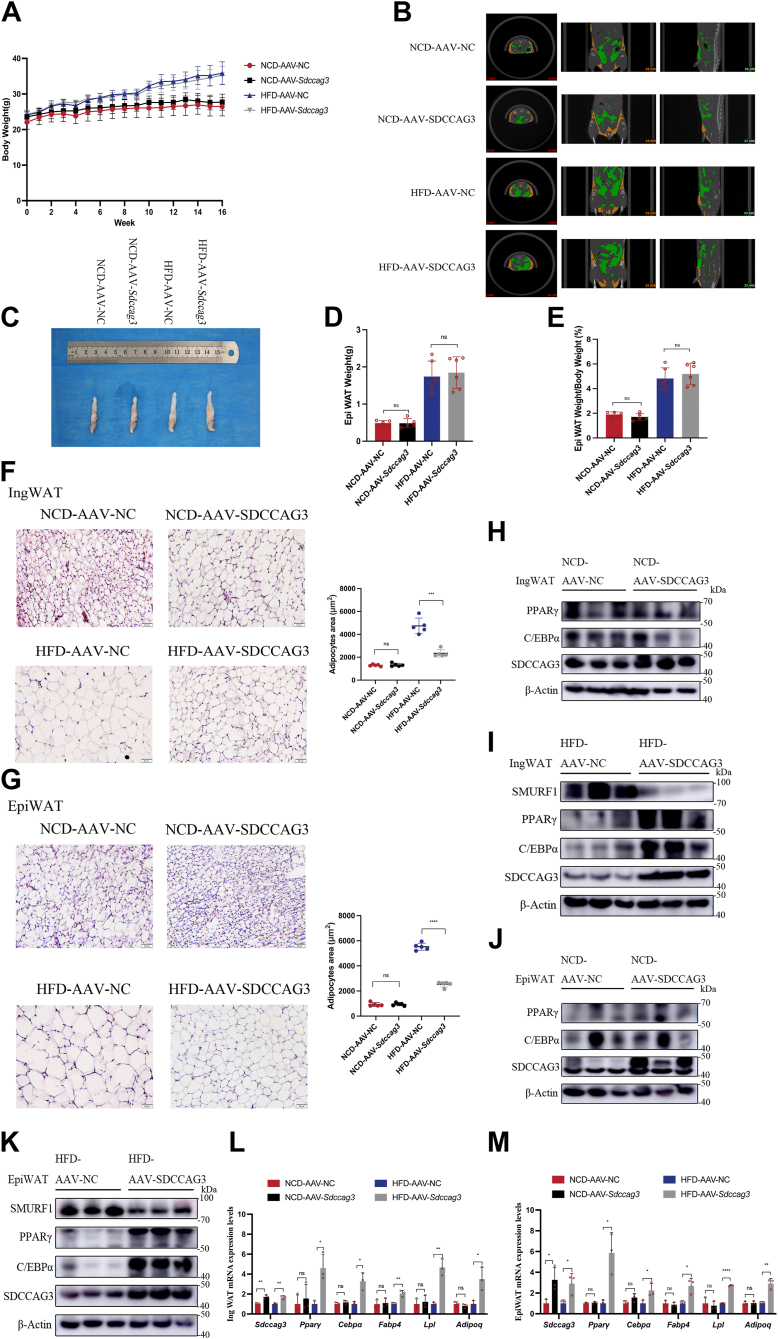
Adipose-specific overexpression of SDCCAG3 inhibited adipocyte hypertrophy and increased the expression of lipid metabolism related indicators. A: Body weight changes in AAV-NC and AAV-*Sdccag3* groups over the course of 16 weeks of NCD or HFD feeding, n = 6. B: Micro-CT scans of AAV-NC and AAV-*Sdccag3* groups of NCD or HFD feeding to reconstruct subcutaneous fat and visceral fat maps. Orange, subcutaneous fat; Green, visceral fat. C: Photos of EpiWAT from AAV-NC and AAV-*Sdccag3* groups of NCD or HFD feeding. D: EpiWAT weight of NCD or HFD-fed AAV-NC and AAV-*Sdccag3* groups, n = 6. E: Ratio of EpiWAT weight to body weight in NCD or HFD-fed AAV-NC and AAV-*Sdccag3* groups, n = 6. F: HE staining of IngWAT from NCD or HFD-fed AAV-NC and AAV-*Sdccag3* groups, scar bar = 50 μm. G: HE staining of EpiWAT from NCD or HFD-fed AAV-NC and AAV-*Sdccag3* groups, scar bar = 50 μm. H: Protein expression levels of PPARγ, C/EBPα and SDCCAG3 in IngWAT of NCD-fed AAV-NC and AAV-*Sdccag3* groups. I: Protein expression levels of PPARγ, C/EBPα, SMURF1 and SDCCAG3 in IngWAT of HFD-fed AAV-NC and AAV-*Sdccag3* groups. J: Protein expression levels of PPARγ, C/EBPα and SDCCAG3 in EpiWAT of NCD-fed AAV-NC and AAV-*Sdccag3* groups. K: Protein expression levels of PPARγ, C/EBPα, SMURF1 and SDCCAG3 in EpiWAT of HFD-fed AAV-NC and AAV-*Sdccag3* groups. L: SDCCAG3, PPARγ, C/EBPα, FABP4, LPL and ADIPOQ mRNA levels in IngWAT of NCD or HFD-fed AAV-NC and AAV-*Sdccag3* groups. M: SDCCAG3, PPARγ, C/EBPα, FABP4, LPL and ADIPOQ mRNA levels in EpiWAT of NCD or HFD-fed AAV-NC and AAV-*Sdccag3* groups. Data were presented as mean ± standard deviation (S.D.). ∗*P* < 0.05, ∗∗*P* < 0.01, ∗∗∗*P* < 0.001, ∗∗∗∗*P* < 0.0001.

**Supplementary Figure S5:**
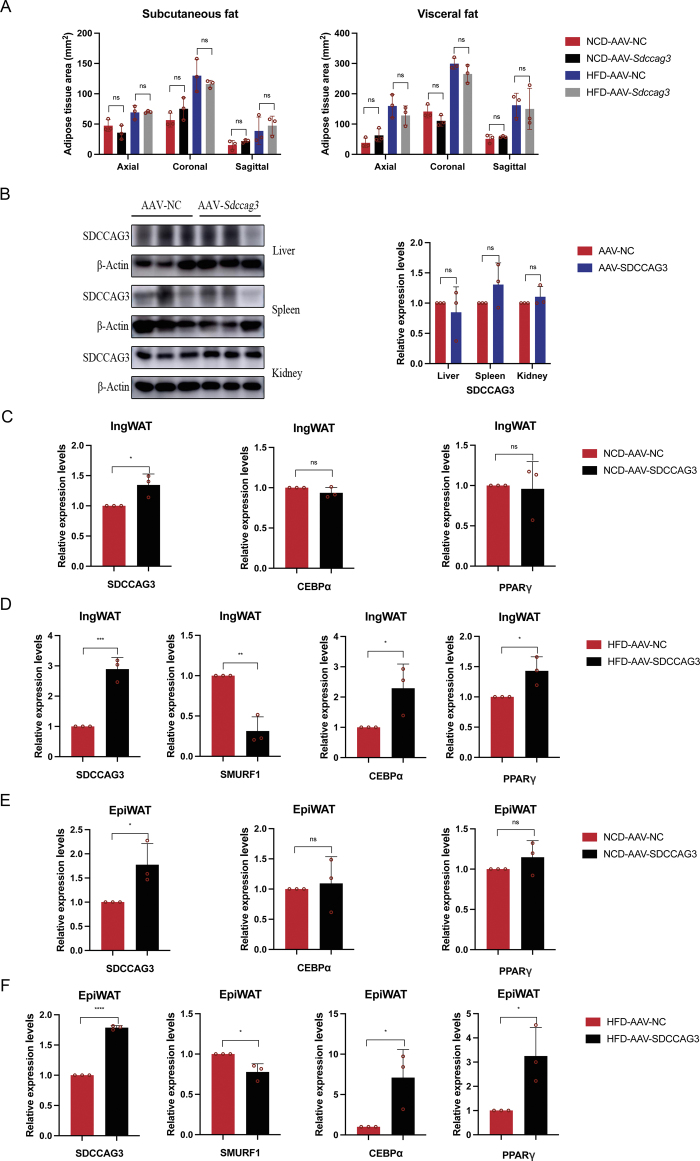


To investigate the promotive effect of SDCCAG3 on adipocyte function in vivo, we constructed an rAAV9-FABP4p-MCS-*Sdccag3*-SV40 overexpression vector and administered it via injection into 4-week-old male C57BL/6J mice. High-fat feeding was initiated two weeks later. During the 16 weeks of either NCD or HFD feeding, adipose-specific overexpression of SDCCAG3 did not significantly alter the body weight of mice on either diet (Fig. 7A). Micro-CT analysis, along with assessments of and EpiWAT weight and the EpiWAT weight/body weight ratio, revealed no significant changes in fat mass in AAV-*Sdccag3* mice compared to AAV-NC mice under both dietary conditions (Fig. 7B–E and supplemental Fig. S5A). Histological analysis and quantitative assessment revealed that adipocyte size in HFD-AAV-*Sdccag3* mice was significantly smaller than that in the control group. In contrast, no significant changes in adipocyte size were observed in the NCD feeding groups (Fig. 7F, G). Additionally, immunoblotting and qPCR analyses showed that the expression of SDCCAG3 was elevated in the IngWAT and EpiWAT of the AAV-*Sdccag3* group compared to the AAV-NC group, with no changes in other tissues, further indicating the specificity of SDCCAG3 overexpression in adipose tissue (supplemental Fig. S5B). In the HFD feeding groups, adipose-specific overexpression of SDCCAG3 promoted the expression levels of lipid metabolism-related factors, including PPARγ, C/EBPα, FABP4, LPL and ADIPOQ, while no changes were observed in the NCD feeding groups. Furthermore, SDCCAG3 overexpression also suppressed the expression level of SMURF1 in vivo (Fig. 7H–M and supplemental Fig. S5C–F). These data suggested that adipose-specific overexpression of SDCCAG3 reduced adipocyte size under high-fat dietary conditions and regulated the expression of multiple genes associated with adipose tissue metabolism, potentially positively influencing metabolic homeostasis in adipose tissue.

### Adipose-specific overexpression of SDCCAG3 increased glucose tolerance and insulin sensitivity and improved systemic metabolism

**Fig. 8:**
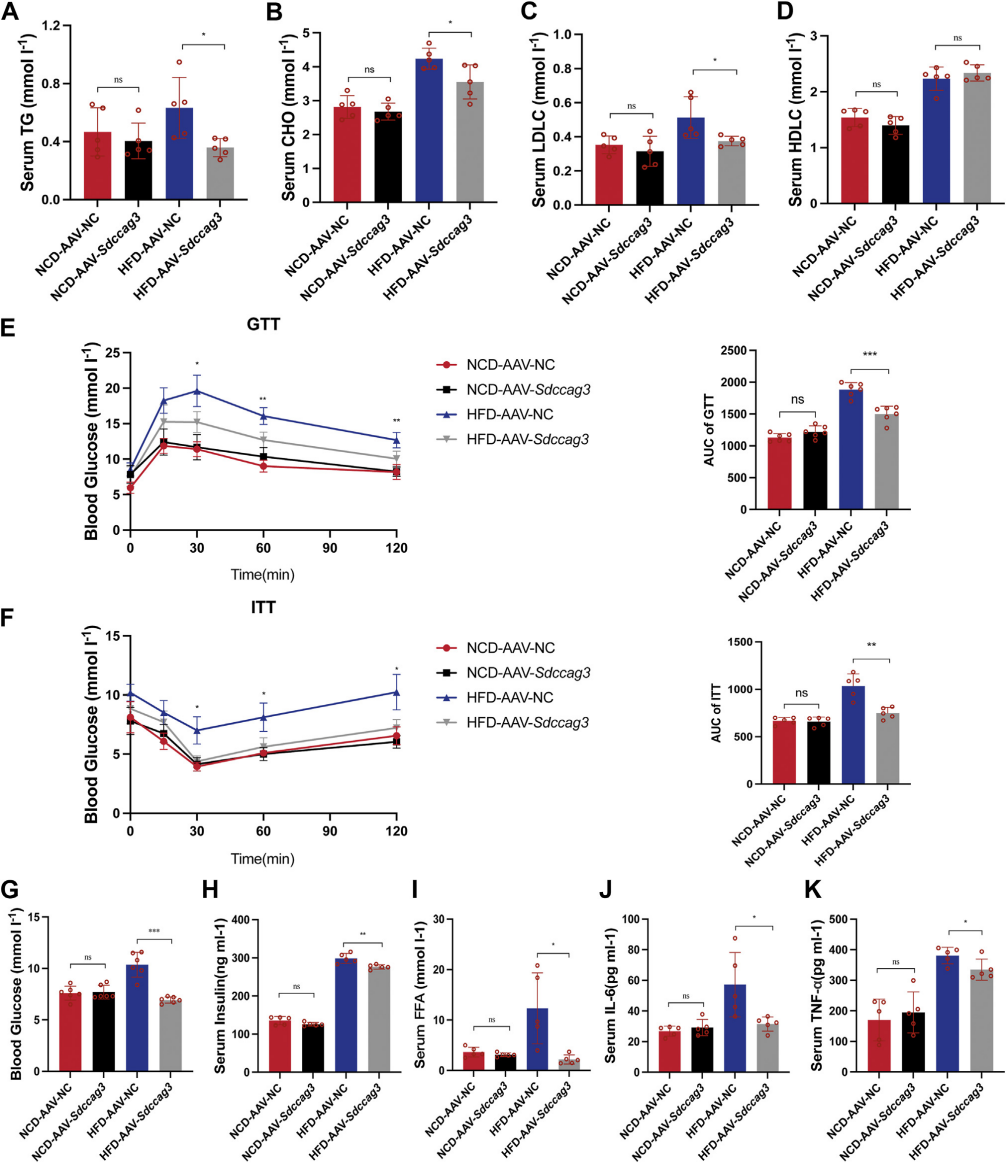
Adipose-specific overexpression of SDCCAG3 improved insulin sensitivity and resisted to obesity-related metabolic disorders. A: Serum triglyceride levels in NCD or HFD-fed AAV-NC and AAV-*Sdccag3* groups, n = 5. B: Serum cholesterol levels in NCD or HFD-fed AAV-NC and AAV-*Sdccag3* groups, n = 5. C: Serum low density lipoprotein cholesterol levels in NCD or HFD-fed AAV-NC and AAV-*Sdccag3* groups, n = 5. D: Serum high density lipoprotein cholesterol levels in NCD or HFD-fed AAV-NC and AAV-*Sdccag3* groups, n = 5. E: Glucose tolerance test and area under curve in NCD or HFD-fed AAV-NC and AAV-*Sdccag3* groups, n = 5. F: Insulin tolerance test and area under curve in NCD or HFD-fed AAV-NC and AAV-*Sdccag3* groups, n = 5. G: Fasting serum glucose levels in NCD or HFD-fed AAV-NC and AAV-*Sdccag3* groups, n = 5. H: Fasting serum insulin levels in NCD or HFD-fed AAV-NC and AAV-*Sdccag3* groups, n = 5. I: Serum free fatty acid levels in NCD or HFD-fed AAV-NC and AAV-*Sdccag3* groups, n = 5. J: Serum IL-6 levels in NCD or HFD-fed AAV-NC and AAV-*Sdccag3* groups, n = 5. K: Serum TNF-α levels in NCD or HFD-fed AAV-NC and AAV-*Sdccag3* groups, n = 5. Data were presented as mean ± standard deviation (S.D.). ∗*P* < 0.05, ∗∗*P* < 0.01, ∗∗∗*P* < 0.001, ∗∗∗∗*P* < 0.0001.

**Supplementary Figure S6:**
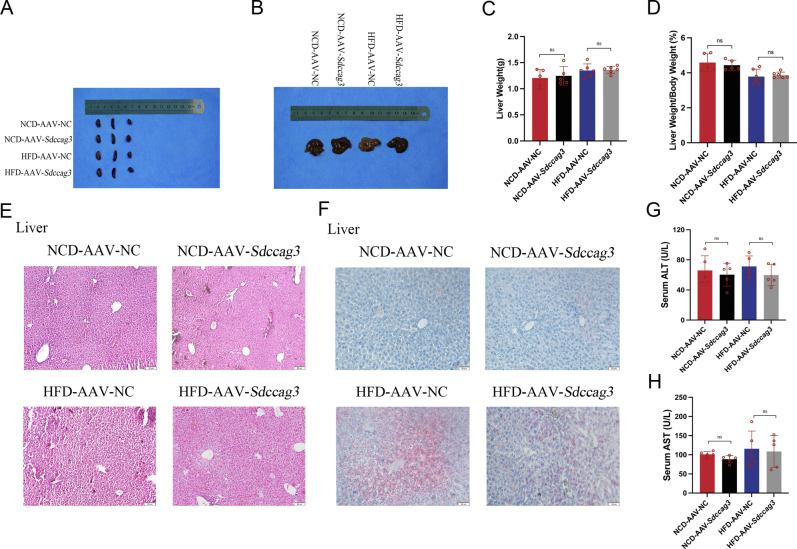


Subsequently, we further examined indicators related to lipid metabolism. In terms of blood lipid levels, HFD-AAV-*Sdccag3* mice showed reduced serum levels of TG, CHO and LDLC, while HDLC levels remained unchanged. And there were no significant changes in NCD feeding groups (Fig. 8A–D). The glucose tolerance test and insulin tolerance test showed that HFD-AAV-*Sdccag3* mice demonstrated improved glucose tolerance and insulin sensitivity compared to HFD-AAV-NC mice, with no changes observed in the NCD feeding groups (Fig. 8E, F). As expected, the HFD led to significant increases in fasting blood glucose, insulin, and FFA levels in mice, whereas adipose-specific overexpression of SDCCAG3 effectively suppressed these increases (Fig. 8G–I). Similarly, adipose-specific overexpression of SDCCAG3 also inhibited the elevation of serum TNF-α and IL-6 levels induced by HFD (Fig. 8J, K). Additionally, no differences were observed in size of the heart, spleen, kidney and liver tissues (supplemental Fig. S6A, B). There were also no differences in liver weight or the liver weight/body weight ratio (supplemental Fig. S6C, D). Hepatic HE and Oil Red O staining indicated that the beneficial effects of SDCCAG3 on adipose tissue under high-fat dietary conditions might reduce hepatic lipid deposition through systemic effects, with no significant differences in ALT and AST (supplemental Fig. S6E–H). These data confirmed that adipose-specific overexpression of SDCCAG3 improved glucose tolerance and insulin sensitivity in mice on HFD, lowered blood lipids, reduced inflammation, and thereby enhanced overall metabolic status.

## Discussion

This study revealed that SDCCAG3 exerted a positive regulatory role in lipid metabolism. This effect was mediated through the attenuation of PPARγ ubiquitination and proteasomal degradation by SDCCAG3, thereby enhancing PPARγ functional activity. Such functionality required synergy with SMURF1 and was regulated by the transcription factor PPARγ, culminating in the formation of the SDCCAG3-PPARγ-SDCCAG3 lipid metabolism enhancement circuit. Additionally, in obese mice, overexpression of SDCCAG3 inhibited adipocyte hypertrophy, alleviated insulin resistance, and improved glucose and lipid levels, while also exhibiting anti-inflammatory properties to some extent, without compromising normal adipose development. Conversely, the deficiency of SDCCAG3 led to more pronounced disruptions in lipid metabolism. This study elucidated the functional role and mechanisms of SDCCAG3 in adipose tissue and metabolism.

We found that the upregulation of SDCCAG3 promoted the expression of PPARγ, C/EBPα, FABP4, LPL and ADIPOQ in adipocytes under a high-fat environment. Research has shown that PPARγ and C/EBPα were considered important regulatory factors for gene transcription in adipocytes, working synergistically to fully activate the expression of genes such as FABP4, LPL and ADIPOQ in mature adipocytes (31). However, in enlarged adipocytes, excessive lipid accumulation inhibited the expression and function of these genes, leading to functional impairments and imbalances in lipid metabolism (32, 33). Therefore, the upregulation of SDCCAG3 promoted the programmed expression of adipose metabolism-related factors, which was beneficial for adipocyte metabolism in a high-fat environment. Moreover, SDCCAG3 inhibited excessive lipid accumulation in adipocytes under high-fat environment. This may be due to improvements in adipocyte metabolism, balancing intracellular lipid storage and breakdown, and preventing excessive lipid accumulation, thereby further contributing to metabolic enhancements (34).

Research has revealed that genes responsible for lipid uptake and metabolism in adipocytes were directly regulated by PPPARγ (15). Furthermore, our previous studies indicated that SDCCAG3 regulated PPARγ in bone tissue of obese mice. Therefore, we investigated the interaction mechanism between SDCCAG3 and PPARγ in adipocytes. We found that SDCCAG3 inhibited the ubiquitination of PPARγ, thereby suppressing its protein degradation through the ubiquitin-proteasome system and enhancing its protein stability. However, studies suggested that SDCCAG3 lacked enzymatic activity (25, 26), indicating the possible presence of intermediate regulatory factor in SDCCAG3's interaction with PPARγ. Among the proteins interacting with SDCCAG3, SMURF1 was known to be an E3 ubiquitin ligase that directly targeted PPARγ as a substrate (30). In this study, we confirmed that SMURF1 served as a key intermediate factor in SDCCAG3's regulation of PPARγ protein and adipocyte function, further elucidating the specific mechanisms through which SDCCAG3 regulated adipocyte function and metabolism.

PPARγ was a hallmark of adiposity-related obesity (35, 36). In obese mice, adipose-specific deletion of SDCCAG3 was found to inhibit the expression of PPARγ and associated metabolic factors, while simultaneously exacerbating adipocyte hypertrophy. Conversely, overexpression of adipose-specific SDCCAG3 promoted the expression of these factors and suppressed adipocyte hypertrophy. This aligned with the characteristic dysfunction of PPARγ in hypertrophic adipocytes, which may be driven by an imbalance in lipid generation and breakdown (17, 18, 34). Furthermore, it was observed that the loss of SDCCAG3 worsened metabolic disturbances associated with obesity in mice, such as impaired glucose tolerance, insulin resistance, elevated lipid levels, and inflammation. Conversely, overexpression of tissue-specific SDCCAG3 improved these obesity-related metabolic disruptions. This was consistent with previous findings indicating that enlarged adipocytes contributed to metabolic disturbances in adipose tissue, thereby impacting the regulation of systemic glucose homeostasis and lipid metabolism. The enlargement of adipocytes served as a crucial predictive indicator of abnormal blood lipid levels and alterations in glucose-insulin homeostasis (32, 37, 38, 39).

Increasingly, studies suggested that the metabolic decline associated with obesity was not solely due to obesity itself, but was linked to the pathological expansion of adipose tissue (9, 40, 41, 42). Our data demonstrated that SDCCAG3 improved metabolism without affecting body weight or adipose tissue weight in obese mice. This may further indicate that the metabolic disruptions related to obesity are independent of obesity metrics and involve pathological interactions between adipose tissue and other organs or systems.

The ability of adipose tissue to expand further relied on the proliferation and hypertrophy of adipocytes (41). In this study, we confirmed that SDCCAG3 regulated the hypertrophy of mature adipocytes. Given that PPARγ is essential for the differentiation of preadipocytes into mature adipocytes (43), we hypothesize that SDCCAG3 might also play a role in the differentiation process of adipocytes by modulating PPARγ. However, whether SDCCAG3 has a dual role in the proliferation and hypertrophy of adipocytes remains to be further investigated. The synergistic effects of these two mechanisms may further enhance the biological function of SDCCAG3 in promoting lipid metabolism.

## Conclusion

This study demonstrated that SDCCGA3 acted as a positive regulator of lipid metabolism, proving that specific overexpression of SDCCGA3 in adipose tissue can improve pathological expansion of adipose tissue and obesity-related metabolic disorders. It further explained that the stability of PPARγ proteins was stabilized through the ubiquitin-proteasome system, which was the key mechanism by which SDCCGA3 promotes adipocyte metabolism. In conclusion, SDCCAG3 exhibited a beneficial regulatory effect on lipid metabolism in the obesity model, potentially providing a novel target for treating obesity-related metabolic diseases and offering a theoretical foundation for the development of clinical therapeutics.

## Data availability

All study data generated or analyzed during this study are included in the article and supplementary information files. Data supporting the findings of this study are available from the corresponding author on reasonable request.

## Supplemental data

This article contains supplemental data.

## Conflict of interest

The authors declare that they have no conflicts of interest with the contents of this article.

## Supplementary data

Supplementary figure legends

Supplementary original blots
