# Supplementary material for: SDCCAG3 inhibits adipocyte hypertrophy and improves obesity-related metabolic disorders via SDCCAG3/SMURF1/PPARγ axis
Source: J Lipid Res. 2025 Mar 7;66(4):100772. doi: 10.1016/j.jlr.2025.100772 (PMC12002885; doi:10.1016/j.jlr.2025.100772)
Supplement: Supplementary Data [file mmc1.doc]

**Supplementary tables**

Table S1. Genotype detection and primers sequence of adipose-specific knockout of *Sdccag3* mice

| **Primers** | **Result pictures** |
| --- | --- |
| Primers1：F1: 5’-GAGACCCACATAAGCAACTGAGTC-3’  R1: 5’-AGGCTACTGTAGGCGATTCAAAG-3’ | **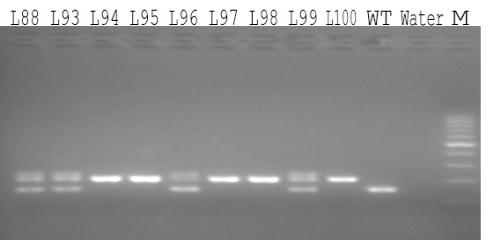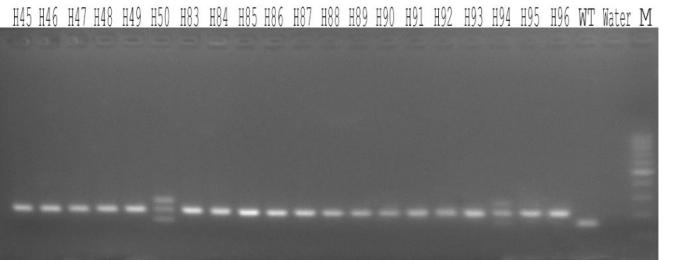** |
| Primers2：Fabp4-Cre-F : 5’-TGATCATTGCCAGGGAGAACCA-3’  Fabp4-Cre-R : 5’-GCGAACATCTTCAGGTTCTGC-3’ | 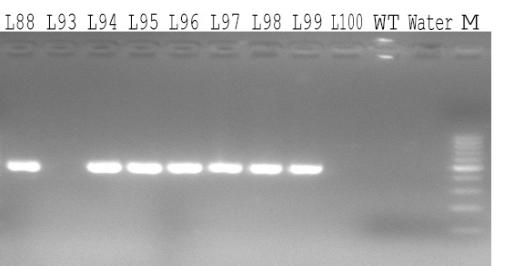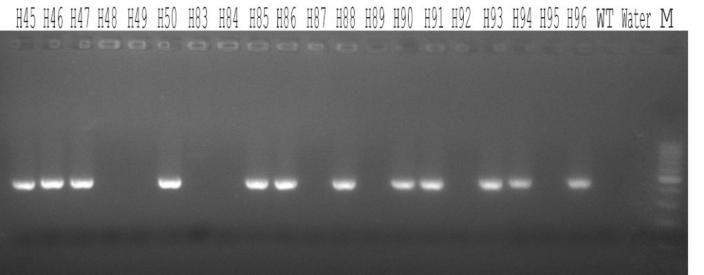 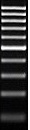 100  300  **500**  **bp**  700  900 |

In terms of primers1, one band with 209bp represented homozygotes, two bands with 209bp and 153bp represented heterozygotes. In terms of primers2, AP2-Cre amplicon was at 473bp

Table S2. The sequence of Primers

| **Primer Name** | **Sequence (5’ to3’)** |
| --- | --- |
| Mus-Smurf1-104F | GCTCAAAGTTCTCAGGCACG |
| Mus-Smurf1-104R | TCTGGCGATACGACTCCTCA |
| Mus-Lpl-135F | TTGGAGAAGCCATCCGTGTG |
| Mus-Lpl-135R | TGTATGCCTTGCTGGGGTTT |
| Mus-Fabp4-173F | GAAAGAAGTGGGAGTGGGCT |
| Mus-Fabp4-173R | TGTCGTCTGCGGTGATTTCAT |
| Mus-Sdccag3-106F | CGGAAGTTGGAGGCGAAGAT |
| Mus-Sdccag3-106R | CAGCCCGTTTGGTCATCAGT |
| Mus-Pparγ-190F | GCTGACCCAATGGTTGCTGA |
| Mus-Pparγ-190R | AGCCTGATGCTTTATCCCCAC |
| Mus-Cebpα-89 F | CCATGCCGGGAGAACTCTAA |
| Mus-Cebpα-89 R | CTCTGGAGGTGACTGCTCATC |
| Mus-Fabp4-90F | TCACCATCCGGTCAGAGAGTA |
| Mus-Fabp4-90R | TCCTGTCGTCTGCGGTGATT |
| Mus-Adipoq-215F | CCGTTCTCTTCACCTACGACC |
| Mus-Adipoq-215R | ATGGGCTATGGGTAGTTGCAG |
| Mus-actin-154F | GGCTGTATTCCCCTCCATCG |
| Mus-actin-154R | CCAGTTGGTAACAATGCCATGT |

Table S3. The sequence of Mus Sdccag3 in pcDNA3.1+

| GCCACC**atg**tcgggctacgcgcggcggcagggcgcgccccctctatcgcggacgcggagccttgtggttcccgacgctcctgcgttctatgagcgccggtcttgtctcccccagctagactgtgagcgcccccatggcggggacctgcacccccatctcttcggctttcggccgacgtttatgtgctatgtgcccagcccggtgctggcttcggtaggagacacaggttttggctatggaaaggggaaatgtactaaccaaggtccttcgggagcccctgagacgcgctttggaggtgacaaacttgaagaccttgaagaagccaatccattctccttcaaagagtttttgaaaaccaagaacctcagcctgtcaaaagaagacacgaccaccagccgaatttacccaaaggaagcctcaaggcacccactgggactagagcacagctcccctgcctcccagctcatgggatatggcctggaatctcagcagccattttttgaagacccaacaagagccagcaacctagaggaggatgaagatgatggatggaatataacctacttgccatctgccgtggatcagactcattcctctagagacacacaggactcaccgccctgtgacacctacctttcctttttctccaactcgtcagagctggcatgtcccgagtctttgcccccatggacgctgagtgacaccgactccaggatctccccagcgtctccagctgggagtcctaatgcagactttgcagctcatgaagaatccctaggggacagacacctgcggacgctgcagataagttatgaagcactgaaagatgaaaactctaagctcagaagaaagctaaatgaggttcagagcttctctgaaactcaaacagaaatggtgaggacactcgaacggaagttggaggcgaagatgatcaaggaggagagtgacttccatgacctcgagtcagtagtccagcaagtcgaacagaaccttgaactgatgaccaaacgggctgtaaaagcagaaaatcatgtcttgaagctgaaacaggaaataaatttgcttcaggcccagctctcaaacttgaggcgagaaaatgaagccctgcggtcaggccagggtgccagcctttctgtagtgaagcagaacaccgacgtggccttgcagaacctccaccttgtcatgaacagtgcacacgcatccataaagcagctggtgtctggggcagacacactgaaccttgttgctgaaatcctcaagtctatcgacagaattagtgaagttaaagatgaggtggactct**-HA-tga** |
| --- |

The full length of the Sdccag3 sequence was divided into three fragments, the sequence after deletion of the corresponding fragments was constructed as mutants separately as follows.

Table S4. The sequence of Mus Sdccag3(△1-145)-HA in pcDNA3.1+

| GCCACC**atg**cacagctcccctgcctcccagctcatgggatatggcctggaatctcagcagccattttttgaagacccaacaagagccagcaacctagaggaggatgaagatgatggatggaatataacctacttgccatctgccgtggatcagactcattcctctagagacacacaggactcaccgccctgtgacacctacctttcctttttctccaactcgtcagagctggcatgtcccgagtctttgcccccatggacgctgagtgacaccgactccaggatctccccagcgtctccagctgggagtcctaatgcagactttgcagctcatgaagaatccctaggggacagacacctgcggacgctgcagataagttatgaagcactgaaagatgaaaactctaagctcagaagaaagctaaatgaggttcagagcttctctgaaactcaaacagaaatggtgaggacactcgaacggaagttggaggcgaagatgatcaaggaggagagtgacttccatgacctcgagtcagtagtccagcaagtcgaacagaaccttgaactgatgaccaaacgggctgtaaaagcagaaaatcatgtcttgaagctgaaacaggaaataaatttgcttcaggcccagctctcaaacttgaggcgagaaaatgaagccctgcggtcaggccagggtgccagcctttctgtagtgaagcagaacaccgacgtggccttgcagaacctccaccttgtcatgaacagtgcacacgcatccataaagcagctggtgtctggggcagacacactgaaccttgttgctgaaatcctcaagtctatcgacagaattagtgaagttaaagatgaggtggactct**-HA-tga** |
| --- |

Table S5. The sequence of Mus Sdccag3(△146-261) –HA in pcDNA3.1+

| GCCACC**atg**tcgggctacgcgcggcggcagggcgcgccccctctatcgcggacgcggagccttgtggttcccgacgctcctgcgttctatgagcgccggtcttgtctcccccagctagactgtgagcgcccccatggcggggacctgcacccccatctcttcggctttcggccgacgtttatgtgctatgtgcccagcccggtgctggcttcggtaggagacacaggttttggctatggaaaggggaaatgtactaaccaaggtccttcgggagcccctgagacgcgctttggaggtgacaaacttgaagaccttgaagaagccaatccattctccttcaaagagtttttgaaaaccaagaacctcagcctgtcaaaagaagacacgaccaccagccgaatttacccaaaggaagcctcaaggcacccactgggactagagctgcggacgctgcagataagttatgaagcactgaaagatgaaaactctaagctcagaagaaagctaaatgaggttcagagcttctctgaaactcaaacagaaatggtgaggacactcgaacggaagttggaggcgaagatgatcaaggaggagagtgacttccatgacctcgagtcagtagtccagcaagtcgaacagaaccttgaactgatgaccaaacgggctgtaaaagcagaaaatcatgtcttgaagctgaaacaggaaataaatttgcttcaggcccagctctcaaacttgaggcgagaaaatgaagccctgcggtcaggccagggtgccagcctttctgtagtgaagcagaacaccgacgtggccttgcagaacctccaccttgtcatgaacagtgcacacgcatccataaagcagctggtgtctggggcagacacactgaaccttgttgctgaaatcctcaagtctatcgacagaattagtgaagttaaagatgaggtggactct**-HA-tga** |
| --- |

Table S6. The sequence of Mus Sdccag3(△262-432) –HA in pcDNA3.1+

| GCCACC**atg**tcgggctacgcgcggcggcagggcgcgccccctctatcgcggacgcggagccttgtggttcccgacgctcctgcgttctatgagcgccggtcttgtctcccccagctagactgtgagcgcccccatggcggggacctgcacccccatctcttcggctttcggccgacgtttatgtgctatgtgcccagcccggtgctggcttcggtaggagacacaggttttggctatggaaaggggaaatgtactaaccaaggtccttcgggagcccctgagacgcgctttggaggtgacaaacttgaagaccttgaagaagccaatccattctccttcaaagagtttttgaaaaccaagaacctcagcctgtcaaaagaagacacgaccaccagccgaatttacccaaaggaagcctcaaggcacccactgggactagagcacagctcccctgcctcccagctcatgggatatggcctggaatctcagcagccattttttgaagacccaacaagagccagcaacctagaggaggatgaagatgatggatggaatataacctacttgccatctgccgtggatcagactcattcctctagagacacacaggactcaccgccctgtgacacctacctttcctttttctccaactcgtcagagctggcatgtcccgagtctttgcccccatggacgctgagtgacaccgactccaggatctccccagcgtctccagctgggagtcctaatgcagactttgcagctcatgaagaatccctaggggacagacac**-HA-tga** |
| --- |

Table S7. Predicted binding sequence and location of Sdccag3 promoter region and transcription factor Pparγ

| >Mus Sdccag3 |
| --- |
| ATATATTAAAGGATGAAACATTGTGTAGAACACTGCAGGCTTGTTTCAGCACTGGCACTCTCCAATGATTGGCACATGAGTGCCCGGCTCTAACAGTGCCTCCCCACAGGAAGTTATAGCCCTTCCAAAGCTCCTCCTGACCACCCAGCCTCAGAGCCACACCTGTCAGTCGGGGGTGCAGAACCACATCAGCCAACAGATCCACCACAGTGTCCAAGCCTTTGCTGTCAGCAGACACAGCATACATGGTGGTGTCTCTGAAAGGAAATAAGCCACAATAGCAGGTGCATGGTTTACACCAGATAACAACTCAGTCACTGAGGCCCTTCATGCCAGCAAAGACACTACCACTACCTCTTTCTTGGGTGTGAAGGCCTTCTGGGAGCCACAACTGCCCACAGTGAAGCTCTCCATGAGATACTTTGGAACCAGAGGAAACTGGTTCTCCTCTCTTCACAGAGGAAGTCTCTCACTCAGGGCTTCCTAGGCCTGGACAAATAACCAGCTGGTCTAAATGAAGACTGGTTTCACATGGACACATGCCCAGCTTCAGATAGCCAGCAGCAGCCTGCCTGTTTAGCTTAGCATTCTTCCCAGGTAAAGGCCTGGACAATTAAAGGTCAAAGGATGTCAGAGAAGAAACTAGTATGGAATGCTTTCTGTCCCCAACATCAGGCATGCTGTGGATTTTCAGAGAGACTCTAAGAGACTCTGACCAAGGATGAGGAGGACATGATGAGGAGGACAGATGGACATGAAATACCTTGAGGTCTGGCAGTCACAGATACCACCATGTTTTTCCAGCGTAAGCAGGATTTCGTCTTTGCTGTCAAATCGAGCAGTGGACTATAAAAATAATTAAGCTTAAGTAAATAACCCAAAATCCATTAAGTGTACATTTGCTTTCAAACACACAAAATCATGTCAGAAGTCTTTTGTGAGCACCAACTTCATATTCAGATCCTTAACTAATACAAGTTCTTAAAAAGGAAAACTGAAAACTTATAAAAATTACAAATTAGACACTGACCGAAAATGCCAATTTTTCCAAAAAATGAGCTATTCCACTAAGATATTTCGCTTCATATCGTGATCCTGAATTAATAAGAACTGGTAATAAAAAGAAAATGGACAGGTTAAGCCAAATATTCAAAACATTTAATCAACAGAAAGTTTTCAAAACAATGGCCAATAGAACTTACTTCCTACGGTACAGAATTGTCCAAACTTATTTTGAGATGCCACACGAAGCCCATTGTCCAGAGTGGTAACTTTGGTTTCAAACTTTTCCTGTCCATCAACTGTTGCAAAAATAGGCTTGGGTACTCCAGGCAAGGGAGAAGAGAGGGGGATGTTGGGGTAGGTGGCACCACTGCTGAACCGTCTGTGTGCAGGAGATCCAAACCTACAGCAGAGCAGCAAAATATTTAGTCTTATTTTATGTCACGGATGTATGTTCATCACAGGCATGCCCGTTGTCCATGGAGGTCAGAAGAGGGCTTTGGATCCCCTGGATAGTGGAGTTATAGATAGATGGTTGTGAGCCACCAAATGGGCGCTGGTAGCCAAACCCAGGGGAAGCAGTCAGTAGGCCTAACTACTGAGCCATCTCTTCGACCCCGGTCTTTGGGGCTTCACTGTCCACTCTGATCAGCAGGCTGCGTGAGAAACGAGTTCCTGTGCATATGGGCCTGGGCAAGCCCGGCCCCTCAGGGCTTCGCTTTGGTCTAAAGGCTCCAGCCGAGGGACTCTGCCGACCACTCCCAACCAGGTTCCAGTCCCCAGCTCCTTCTCCAGGCCCAAGGCGCCTGTGCTCTGATCCAGAGGCCCATTCTTCTCTTCCAGTTCCCCCCGGTACCAACTCCCCCGAGTCCTCCCTTCCTCGCACCGAGACAGGCCTCGGCTCACCTGGGCCGCGCGCACAGCACCGCAGATCCCCGAAGCAGCCGCGCAGCCGCCCACACTGCCGTCGCCATCGCGCCGCCGCGCTTCTGCGGTCACTTCCGCTTCCGACCCCGCCCCGCGGCGTTCGGCGCTGGCTTCCGGCCCGCCGAGCCGGATGAGGCAGGGGCCGGCGACTGCCTTGCGGGCCATGGGGCCGCGGCCGGGTTAGGGCGCGGCGATGTCGGGCTACGCGCGGCGGCAGGGCGCGCCCCCTCTATCGCGGACGCGGAGCCTTGTGGTTCCCGACGGT |

The underlined part was the sequence of the Sdccag3 luciferase reporter gene plasmid constructed. And the region deleted the yellow marked sequence was the part for the construction of Sdccag3 mutant luciferase reporter gene plasmid.
