# Supplementary material for: SDCCAG3 inhibits adipocyte hypertrophy and improves obesity-related metabolic disorders via SDCCAG3/SMURF1/PPARγ axis
Source: J Lipid Res. 2025 Mar 7;66(4):100772. doi: 10.1016/j.jlr.2025.100772 (PMC12002885; doi:10.1016/j.jlr.2025.100772)
Supplement: Supplementary figure legends [file mmc2.docx]

**Figure S1 Quantitative data from western blot analyses.**

1. Quantitative analysis of SDCCAG3 protein expression level in IngWAT of mice on NCD or HFD. **B)** Quantitative analysis of SDCCAG3 protein expression level in EpiWAT of mice on NCD or HFD. **C)** Quantitative analysis of SDCCAG3 protein expression level during the differentiation of 3T3-L1 cells into mature adipocytes. **D)** Quantitative analysis of SDCCAG3 protein expression level in differentiated 3T3-L1 cells cultured under normal or high-fat conditions. **E)** Quantitative analysis of protein expression levels of PPARγ, C/EBPα and SDCCAG3 with knockdown or overexpression of SDCCAG3 on differentiated 3T3-L1 cells cultured under high-fat conditions. **F)** Quantitative analysis of Flag-PPARγ and SDCCAG3 protein expression levels in HEK293T cells with CHX treatment and MG132 or CHQ dual treatment for 4h. **G)** Quantitative analysis of SDCCAG3 protein expression level and ubiquitination level of PPARγ in HEK293T cells after knockdowning or overexpressing SDCCAG3. **H)** Quantitative analysis of protein expression level of SMURF1 with knockdown or overexpression of SDCCAG3 on differentiated 3T3-L1 cells. **I)** Quantitative analysis of ubiquitination level of PPARγ in HEK293T cells with overexpression of SDCCAG3 and SMURF1.

**Figure S2 Quantitative data from western blot analyses.**

1. Quantitative analysis of protein expression levels of SDCCAG3, SMURF1, PPARγ and C/EBPα in differentiated 3T3-L1 cells with overexpression of SDCCAG3 and SMURF1. **B)** Quantitative analysis of protein expression levels of SDCCAG3, PPARγ and C/EBPα in differentiated 3T3-L1 cells with overexpressing SDCCAG3 and GW9662 treatment. **C)** Quantitative analysis of protein expression levels of SDCCAG3, PPARγ and C/EBPα in differentiated 3T3-L1 cells with transfection of full-length SDCCAG3 and mut1, mut2, mut3 mutants. **D)** Quantitative analysis of protein expression levels of SDCCAG3 and PPARγ in differentiated 3T3-L1 cells with overexpressing PPARγ.

**Figure S3 Quantitative data from adipose-specific knockout of SDCCAG3 analyses.**

1. Quantitative analysis of micro-CT scans of AKO-WT and AKO-*Sdccag3* groups of NCD or HFD feeding. **B)** Protein expression levels of SDCCAG3 in liver, spleen and kidney tissues of NCD or HFD-fed AKO-WT and AKO-*Sdccag3* groups. **C)** Quantitative analysis of protein expression levels of PPARγ, C/EBPα and SDCCAG3 in IngWAT of NCD-fed AKO-WT and AKO-*Sdccag3* groups. **D)** Quantitative analysis of protein expression levels of SMURF1, PPARγ, C/EBPα and SDCCAG3 in IngWAT of HFD-fed AKO-WT and AKO-*Sdccag3* groups. **E)** Quantitative analysis of protein expression levels of PPARγ, C/EBPα and SDCCAG3 in EpiWAT of NCD-fed AKO-WT and AKO-*Sdccag3* groups. **F)** Quantitative analysis of protein expression levels of SMURF1, PPARγ, C/EBPα and SDCCAG3 in EpiWAT of HFD-fed AKO-WT and AKO-*Sdccag3* groups.

**Figure S4 Effect of adipose-specific knockout of SDCCAG3 on the liver.**

1. Photos of heart, spleen and kidney tissues from AKO-WT and AKO-*Sdccag3* groups of NCD or HFD feeding. B) Photos of liver from AKO-WT and AKO-*Sdccag3* groups of NCD or HFD feeding. C) Liver weight of NCD or HFD-fed AKO-WT and AKO-*Sdccag3* groups, n=5. D) Ratio of liver weight to body weight in NCD or HFD-fed AKO-WT and AKO-*Sdccag3* groups, n=5. E) HE staining of liver from NCD or HFD-fed AKO-WT and AKO-*Sdccag3* groups, bar=50μm. F) Oil red O staining of liver from NCD or HFD-fed AKO-WT and AKO-*Sdccag3* groups, bar=50μm. G) Serum ALT levels in NCD or HFD-fed AKO-WT and AKO-*Sdccag3* groups, n=5. H) Serum AST levels in NCD or HFD-fed AKO-WT and AKO-*Sdccag3* groups, n=5.

**Figure S5 Quantitative data from adipose-specific overexpression of SDCCAG3 analyses.**

1. Quantitative analysis of micro-CT scans of AAV-NC and AAV-*Sdccag3* groups of NCD or HFD feeding. **B)** Protein expression levels of SDCCAG3 in liver, spleen and kidney tissues of NCD or HFD-fed AAV-NC and AAV-*Sdccag3* groups. **C)** Quantitative analysis of protein expression levels of PPARγ, C/EBPα and SDCCAG3 in IngWAT of NCD-fed AAV-NC and AAV-*Sdccag3* groups. **D)** Quantitative analysis of protein expression levels of SMURF1, PPARγ, C/EBPα and SDCCAG3 in IngWAT of HFD-fed AAV-NC and AAV-*Sdccag3* groups. **E)** Quantitative analysis of protein expression levels of PPARγ, C/EBPα and SDCCAG3 in EpiWAT of NCD-fed AAV-NC and AAV-*Sdccag3* groups. **F)** Quantitative analysis of protein expression levels of SMURF1, PPARγ, C/EBPα and SDCCAG3 in EpiWAT of HFD-fed AAV-NC and AAV-*Sdccag3* groups.

**Figure S6 Effect of adipose-specific overexpression of SDCCAG3 on the liver.**

**A)** Photos of heart, spleen and kidney tissues from AAV-NC and AAV-*Sdccag3* groups of NCD or HFD feeding. **B)** Photos of liver from AAV-NC and AAV-*Sdccag3* groups of NCD or HFD feeding. **C)** Liver weight of NCD or HFD-fed AAV-NC and AAV-*Sdccag3* groups, n=6. **D)** Ratio of liver weight to body weight in NCD or HFD-fed AAV-NC and AAV-*Sdccag3* groups, n=6. **E)** HE staining of liver from NCD or HFD-fed AAV-NC and AAV-*Sdccag3* groups, scar bar=50μm. **F)** Oil red O staining of liver from NCD or HFD-fed AAV-NC and AAV-*Sdccag3* groups, scar bar=50μm. **G)** Serum ALT levels in NCD or HFD-fed AAV-NC and AAV-*Sdccag3* groups, n=5. **H)** Serum AST levels in NCD or HFD-fed AAV-NC and AAV-*Sdccag3* groups, n=5.
